# Addressing personal protective equipment (PPE) decontamination: Methylene blue and light inactivates severe acute respiratory coronavirus virus 2 (SARS-CoV-2) on N95 respirators and medical masks with maintenance of integrity and fit

**DOI:** 10.1017/ice.2021.230

**Published:** 2022-07

**Authors:** Thomas Sean Lendvay, James Chen, Brian H. Harcourt, Florine E. M. Scholte, Ying Ling Lin, F. Selcen Kilinc-Balci, Molly M. Lamb, Kamonthip Homdayjanakul, Yi Cui, Amy Price, Belinda Heyne, Jaya Sahni, Kareem B. Kabra, Yi-Chan Lin, David Evans, Christopher N. Mores, Ken Page, Larry F. Chu, Eric Haubruge, Etienne Thiry, Louisa F. Ludwig-Begall, Constance Wielick, Tanner Clark, Thor Wagner, Emily Timm, Thomas Gallagher, Peter Faris, Nicolas Macia, Cyrus J. Mackie, Sarah M. Simmons, Susan Reader, Rebecca Malott, Karen Hope, Jan M. Davies, Sarah R. Tritsch, Lorène Dams, Hans Nauwynck, Jean-Francois Willaert, Simon De Jaeger, Lei Liao, Mervin Zhao, Jan Laperre, Olivier Jolois, Sarah J. Smit, Alpa N. Patel, Mark Mayo, Rod Parker, Vanessa Molloy-Simard, Jean-Luc Lemyre, Steven Chu, John M. Conly, May C. Chu

**Affiliations:** 1 Department of Urology, University of Washington School of Medicine, Seattle Children’s Hospital, Seattle, Washington, United States; 2 Viral Special Pathogens Branch, Division of High Consequence Pathogens and Pathology, National Center for Emerging and Zoonotic Infectious Diseases, Centers for Disease Control and Prevention, Atlanta, Georgia, United States; 3 Department of Infectious Diseases, Microbiology and Immunology, CRCHU de Québec-Université Laval, Québec, Québec, Canada; 4 World Health Organization, Geneva, Switzerland; 5 National Personal Protective Technology Laboratory (NPPTL), National Institute for Occupational Safety and Health (NIOSH), Centers for Disease Control and Prevention (CDC), Pittsburgh, Pennsylvania, United States; 6 Department of Epidemiology, Colorado School of Public Health, Anschutz Medical Campus, Aurora, Colorado, United States; 7 Center for Global Health, Colorado School of Public Health, Anschutz Medical Campus, Aurora, Colorado, United States; 8 Department of Materials Science and Engineering, Stanford University, Stanford, California, United States; 9 The Anesthesia, Informatics and Media (AIM) Lab, Stanford University School of Medicine, Stanford, California, United States; 10 Department of Chemistry, University of Calgary, Calgary, Alberta, Canada; 11 Seattle Children’s Research Institute, Seattle, Washington, United States; 12 Department of Global Health, Milken Institute School of Public Health, The George Washington University, Washington, DC, United States; 13 Department of Medical Microbiology and Immunology, University of Alberta, Edmonton, Alberta, Canada; 14 Department of Medical Microbiology and Immunology, University of Alberta, Edmonton, Alberta, Canada; 15 Alberta Health Services, Alberta, Canada; 16 Gembloux AgroBioTech, Terra Research Center, University of Liège, Gembloux, Belgium; 17 Department of Infectious and Parasitic Diseases, Faculty of Veterinary Medicine, University of Liège, Liège, Belgium; 18 Department of Radiology, University of Washington School of Medicine, Seattle, Washington, United States; 19 Department of Microbiology and Immunology, Loyola University Chicago, Maywood, Illinois, United States; 20 W21C Research and Innovation Centre, University of Calgary, Calgary, Alberta, Canada; 21 Department of Anesthesiology, Perioperative and Pain Medicine, University of Calgary, Calgary, Alberta, Canada; 22 Faculty of Veterinary Medicine, Ghent University, Merelbeke, Belgium; 23 4CAir, Inc, Sunnyvale, California, United States; 24 Centexbel, Grace-Hollogne, Belgium; 25 Nelson Laboratories, Salt Lake City, Utah, United States; 26 British Standards Institution, London, United Kingdom; 27 Stryker, Québec, Québec, Canada; 28 Department of Physics, Molecular and Cellular Physiology, Stanford University, Stanford, California, United States

## Abstract

**Objective:**

The coronavirus disease 2019 (COVID-19) pandemic has resulted in shortages of personal protective equipment (PPE), underscoring the urgent need for simple, efficient, and inexpensive methods to decontaminate masks and respirators exposed to severe acute respiratory coronavirus virus 2 (SARS-CoV-2). We hypothesized that methylene blue (MB) photochemical treatment, which has various clinical applications, could decontaminate PPE contaminated with coronavirus.

**Design:**

The 2 arms of the study included (1) PPE inoculation with coronaviruses followed by MB with light (MBL) decontamination treatment and (2) PPE treatment with MBL for 5 cycles of decontamination to determine maintenance of PPE performance.

**Methods:**

MBL treatment was used to inactivate coronaviruses on 3 N95 filtering facepiece respirator (FFR) and 2 medical mask models. We inoculated FFR and medical mask materials with 3 coronaviruses, including SARS-CoV-2, and we treated them with 10 µM MB and exposed them to 50,000 lux of white light or 12,500 lux of red light for 30 minutes. In parallel, integrity was assessed after 5 cycles of decontamination using multiple US and international test methods, and the process was compared with the FDA-authorized vaporized hydrogen peroxide plus ozone (VHP+O_3_) decontamination method.

**Results:**

Overall, MBL robustly and consistently inactivated all 3 coronaviruses with 99.8% to >99.9% virus inactivation across all FFRs and medical masks tested. FFR and medical mask integrity was maintained after 5 cycles of MBL treatment, whereas 1 FFR model failed after 5 cycles of VHP+O_3_.

**Conclusions:**

MBL treatment decontaminated respirators and masks by inactivating 3 tested coronaviruses without compromising integrity through 5 cycles of decontamination. MBL decontamination is effective, is low cost, and does not require specialized equipment, making it applicable in low- to high-resource settings.

The coronavirus disease 2019 (COVID-19) pandemic caused by severe acute respiratory coronavirus virus 2 (SARS-CoV-2) has resulted in critical personal protective equipment (PPE) shortages, especially filtering facepiece respirators (FFRs, also known as N95 respirators). Although designed for single-use, healthcare personnel (HCP) are reusing potentially contaminated FFRs and medical masks (MMs) on an emergency basis due to supply shortages. These shortages have necessitated the rapid development and deployment of decontamination processes, leading to the World Health Organization (WHO) issuing interim guidance on rational PPE use.^1^ The US Food and Drug Administration (FDA) granted emergency use authorization of hydrogen peroxide and steam sterilization systems to decontaminate FFRs for reuse.^2,3^ These technologies remain less available in low-resource settings, where frontline HCP have inadequate protection^4,5^; thus, novel methods for PPE decontamination are needed.

Photochemical disinfection uses a photosensitive chemical that, combined with visible light, generates singlet oxygen (Supplementary Material online). Singlet oxygen inactivates viruses by damaging viral nucleic acids and membranes.^6^ One such photosensitizer is methylene blue (MB), which is FDA-approved to treat methemoglobinemia and is used to sterilize human plasma transfusions in Europe.^7^ MB inactivates SARS-CoV-2 and many other viruses (Supplementary Table S3 online).^8–10^

In this Development and Methods for N95 Respirators and Mask Decontamination (DeMaND) study, we evaluated methods that inactivate SARS-CoV-2 on respirators and masks that can be applied anywhere at low cost. We sought to determine whether MB with light (MBL) could effectively decontaminate commonly used FFRs and medical masks while maintaining mask integrity (i.e., filtration, breathability, fluid resistance, and fit) after multiple decontamination cycles. We leveraged 4 virology laboratories and 6 PPE integrity testing sites to examine the MBL virucidal effect on 2 SARS-CoV-2 isolates (betacoronavirus) and 2 coronaviruses requiring a lower level of biocontainment (the betacoronavirus murine hepatitis virus (MHV) and the alphacoronavirus porcine respiratory coronavirus (PRCV)) and (2) to determine the impact of 5 cycles of decontamination on PPE integrity (Fig. 1).

**Fig. 1.**
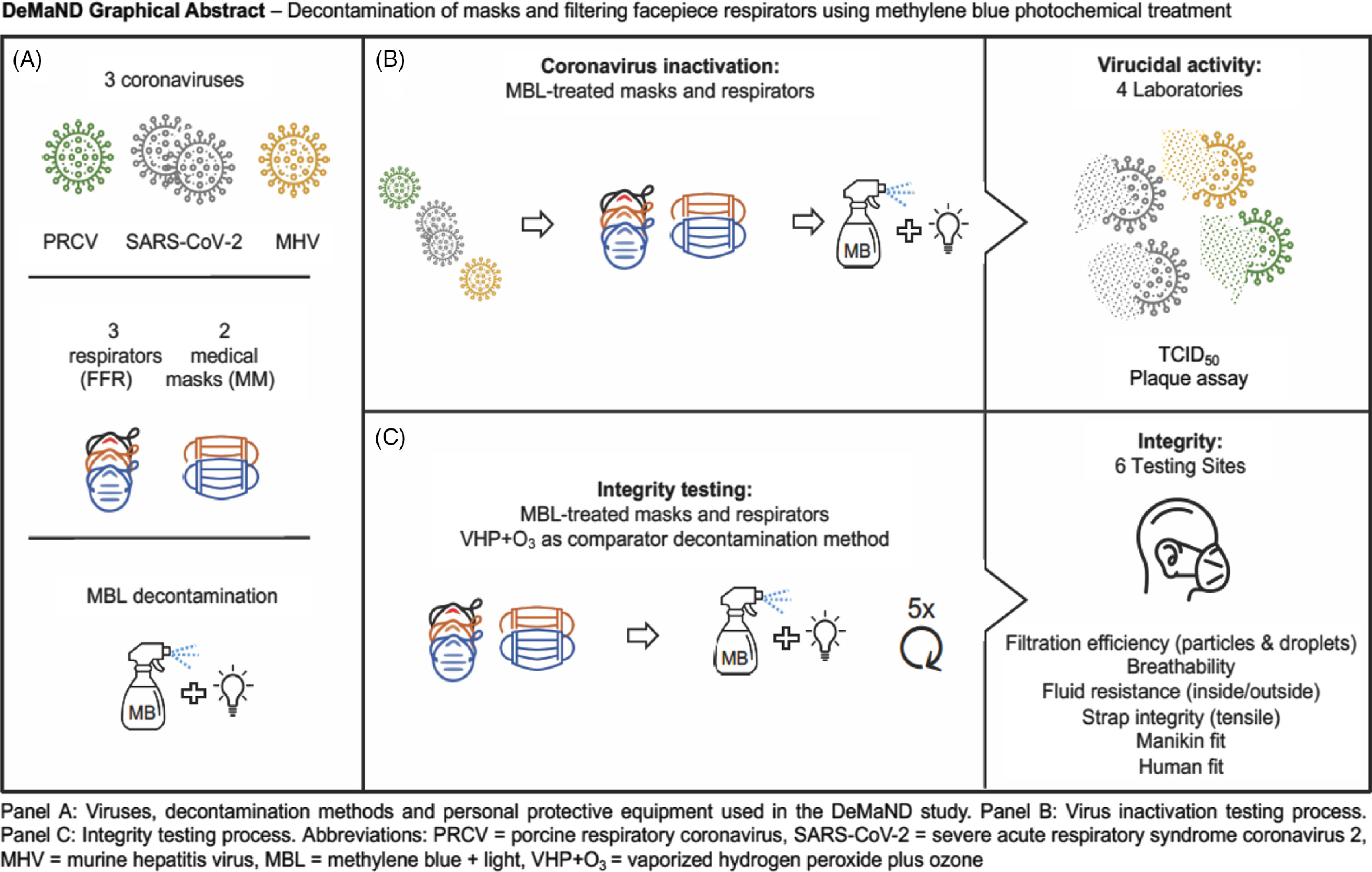
Graphical representation of the DeMaND study methodology. (A) Overview of the coronaviruses, respirators, masks, and decontamination methods used. (B) FFRs and medical masks were inoculated with virus and treated with MBL. The remaining infectious virus was quantified using TCID_50_ or plaque assay. (C) In parallel with the virucidal testing of MBL, intact FFRs and medical masks were subjected to 5 cycles of decontamination before mask integrity was tested using the indicated methods. Note. FFR, filtering facepiece respirator; PRCV, porcine respiratory coronavirus; SARS-CoV-2, severe acute respiratory syndrome coronavirus 2; MHV, murine hepatitis virus; MBL, methylene blue + light; VHP+O_3_, vaporized hydrogen peroxide plus ozone. See Supplemental Table S1 (online) for the respirator and mask decontamination and testing matrix.

We selected the number of decontamination cycles based on the Centers for Disease Control and Prevention (CDC)’s recommended maximum number of donnings as part of crisis capacity strategies at the time this study was conducted.^11^ We chose FFRs and medical masks based on availability during recent outbreak responses and variations in shape, material, and structure. For integrity testing, we compared MBL to the FDA-authorized vaporized hydrogen peroxide plus ozone system (VHP+O_3_).^2^

## Methods

### Respirators and masks

We tested 3 FFR and 2 medical mask models, both fluid-resistant and non–fluid-resistant (Supplementary Fig. S9 online). These FFRs are surgical FFRs which are National Institute of Occupational Safety and Health (NIOSH)–approved particulate respirators cleared by FDA as medical devices (Supplementary Table S1 testing matrix online).

### Viruses

We obtained SARS-CoV-2 isolates from a patient at the George Washington University Hospital (Lab 1) and from Dr. Darryl Falzarano at the Vaccine and Infectious Disease Organization (Lab 2; GISAID accession no. EPI_ISL_425177). We used a SARS-CoV-2 clinical sputum specimen with University of Calgary Conjoint Health Research Ethics Board approval (no. REB20-0444). Recombinant MHV strain rA59-E-FL-M and PRCV strain 91V44 have been described previously.^12–15^

### Virus inoculation and elution

We cut FFRs and medical masks into 7×10-mm coupons and inoculated them with the maximum available virus dose of SARS-CoV-2 or MHV on the outer layer (or inner layer where specified) with a pipette and dried them for 20 minutes before treatment. Virus was eluted in media using a vortexer or orbital rocker. Alternatively, we injected PRCV under the outer layer, then we excised 34×34-mm coupons and eluted using a vortexer. We quantified the remaining infectious virus by median tissue culture infectious dose (TCID_50_) or plaque assays.

### Methylene blue treatment

We added 10 µM MB to the inoculated coupons or sprayed it on intact inoculated masks. We exposed them to 50,000 lux of white light or 12,500 lux of red light for 30 minutes. We used red light at a lower intensity because red light contains a higher percentage of wavelengths that activate MB (Supplementary Fig. S1 online). Dark controls were left in the biosafety cabinet with the light off or were covered with aluminum foil (<100 lux).

We soaked 3M Panel respirator (model 1870+, hereafter R3) coupons with 10 µM MB for >1 hour and dried them for 2 days protected from light for pretreatment testing. We then spotted SARS-CoV-2 on outer or inner mask layers and dried these coupons for 20 minutes before exposing them to 50,000 lux of white light for 30 minutes. Intact 3M half-sphere respirator (model 1860, hereafter RM) and Type II RASTM F2100 Level 2 Halyard face mask (hereafter FH) were sprayed with 7–8 mL 10 µM MB and dried overnight. We added MHV to 3 points on the outer surface, dried for 20 minutes and exposed them to light (50,000 lux) for 30 minutes. We then excised the inoculated area, eluted, and quantified the virus by TCID_50_ assay.

### Light sources

The Seattle Children’s Research Institute, George Washington University, University of Calgary, and Nelson Laboratories used lightboxes developed at Colorado State University that included 4000K Husky LED lights. The University of Alberta used 3500K Husky LEDs in their lightboxes. The University of Liège and Centexbel used a custom lightbox containing horticultural lamps. All laboratories verified light intensity using light meters (Supplementary Methods online).

### Integrity testing

We assessed FFR and medical mask integrity by determining filtration efficiency, breathability, fluid resistance, and fit. We tested FFRs and medical masks untreated and after 5 cycles of decontamination with VHP+O_3_ or 10 µM MB plus white light (50,000 lux) or red light (12,500 lux) for 60 minutes. For the VHP+O_3_ treatment, we used the preset cycle (cycle 1) of the Sterizone VP4 Sterilizer (Stryker, Québec, Québec, Canada) at 41°C.

#### Filtration efficiency testing

We assessed filtration efficiency using NaCl submicron charged-neutralized particles ranging in size from 0.022 to 0.259 µm with a median count diameter of 0.075 ± 0.020 µm and a geometric standard deviation of <1.86 to give a mass median aerodynamic diameter of 0.3 μm, with airflow at 85 L/minute (simulating inhalation at heavy workload).^16^ We measured bacterial filtration efficiency of medical masks using aerosolized droplets containing *Staphylococcus aureus* at a 28.3 L/minute air flow rate (Supplementary Methods and Results online).^17^

#### Breathability testing

We assessed breathability by measuring inhalation and exhalation breathing resistances using standard test methods,^18,19^ and we used pressure-drop measurements for medical masks.^17^ Additionally, we determined Sheffield Dummy airflow differences for both FFRs and medical masks (Supplemental Methods and Results online).

#### Fluid resistance testing

Testing of resistance to splash and spray by synthetic blood is required for surgical masks in the United States and fluid-resistant medical masks in Europe. We tested fluid resistance for medical masks (Supplemental Methods and Results online).

#### Fit testing

We conducted human fit testing with the PortaCount Pro+ 8038 (TSI, Shoreview, MN). Fit testing was exempted from ethics board review by both the Research Compliance Office of Stanford University and the Conjoint Health Research Ethics Board of the University of Calgary. We tested multiple dynamic tasks: regular breathing, heavy breathing, turning head side-to-side, moving head up-and-down, talking, and bending over while breathing. We performed each set of tests twice and calculated a fit factor for each mask. According to the NIOSH/National Personal Protective Technology Laboratory (NPPTL) Decontaminated Respirator Assessment Plan, we conducted manikin fit testing using an advanced, realistic manikin head.^16^ We examined the changes in the elastic recovery of the FFR straps and medical-mask ear loops to determine strap and ear-loop integrity changes after 5 cycles of decontamination (Supplementary Methods and Results online).

#### Statistical analysis

We calculated means and standard deviations or percentage passing of each integrity test method separately by FFR or medical mask model. We combined data for integrity test methods conducted at >1 test site to create overall means and standard deviations or percentage passing. We tested the normality of the data distribution using the Shapiro-Wilk test. We calculated significant differences between untreated and treated FFRs and medical masks using the Student *t-test*, the Mann-Whitney U test, or the Fisher exact test, as appropriate. We used SAS version 9.4 software (SAS Institute, Cary, NC) for the analysis.

## Results

### Methylene blue and light (MBL) tissue culture plate inactivation

**Fig. 2.**
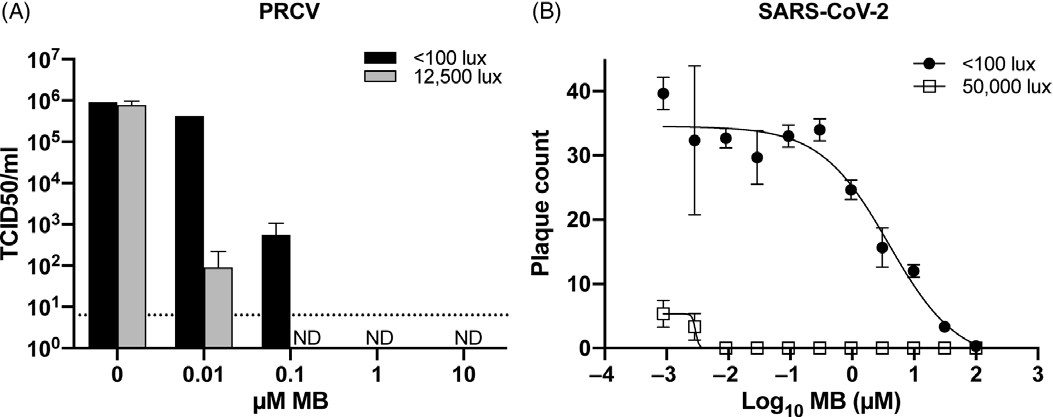
Inactivation of PRCV and SARS-CoV-2 using MBL. (A) To determine the efficacy of different MB concentrations, we added serial dilutions of MB to wells of a 48-well plate containing 10 µL PRCV (2×10^7^ TCID_50_/ml). Plates were either exposed to red light (12,500 lux) for 30 minutes or were protected from light (<100 lux). The dotted line indicates the limit of detection. (B) We added serial dilutions of MB to wells of a 12-well plate containing ~50 PFU SARS-CoV-2 in MEM plus 15% FCS. Plates were either exposed to white light (50,000 lux) for 45 minutes or protected from light (<100 lux). We determined viral titers using 2–3 replicate samples. Note. FCS, fetal calf serum; MEM, minimum essential media, ND, not detected.

We confirmed that MBL can inactivate a coronavirus with varying concentrations of MB when mixed with PRCV and exposed to red light (12,500 lux). Treatment with 0.1 µM MB plus light resulted in complete inactivation. In the absence of additional light, complete inactivation required a dose of 1 µM MB (Fig. 2A).

**Fig. 3.**
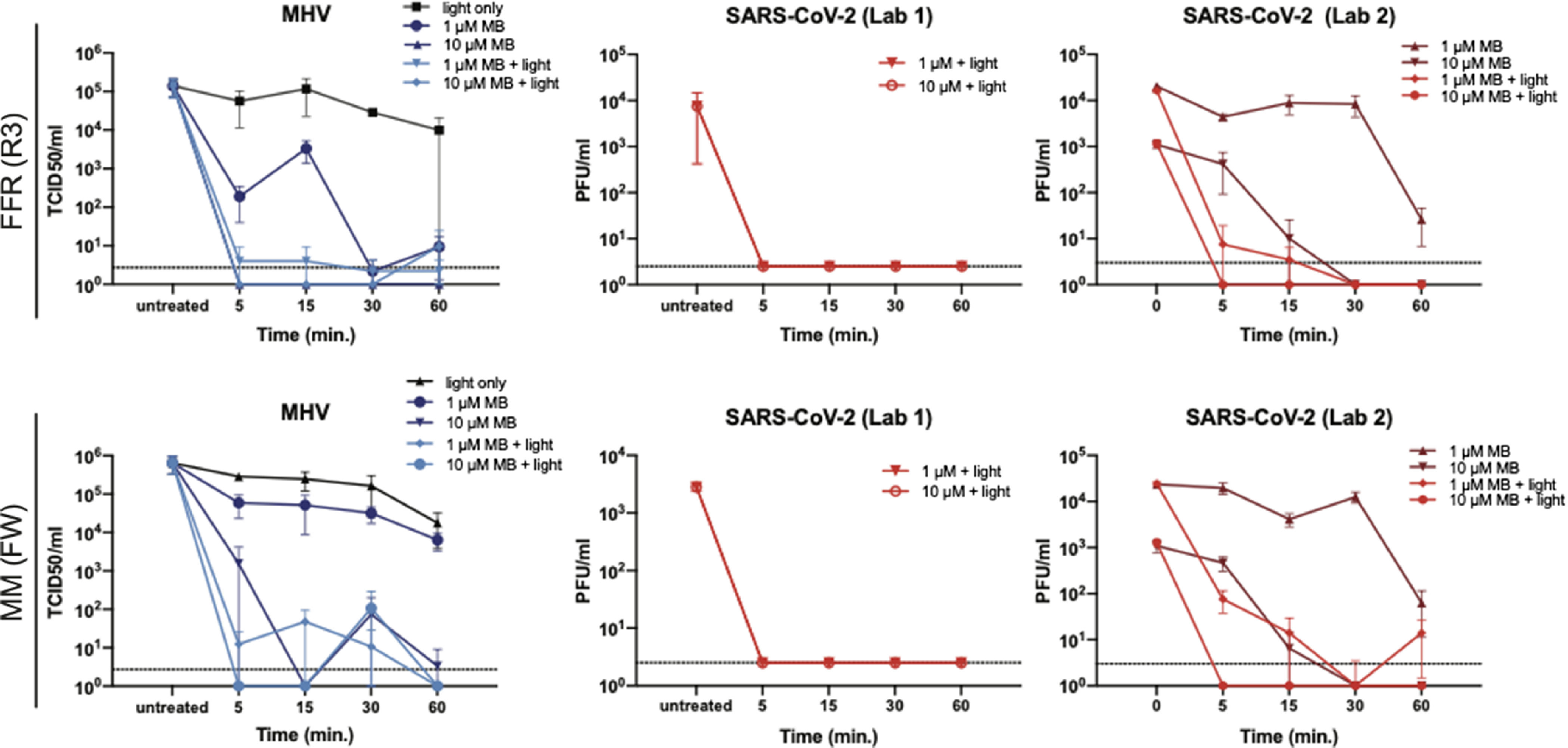
MBL inactivates MHV and SARS-CoV-2 on FFR and medical mask material. (A) Effect of MBL treatment on MHV and SARS-CoV-2 titers. We applied a 10-µL aliquot of MHV or SARS-CoV-2 to coupons derived from an FFR (R3) or medical mask (FW) and they were left to dry for 20 minutes. Subsequently, we added 10 or 30 µL MB to each coupon at the indicated concentrations. We exposed the samples to white light (50,000 lux) for the indicated periods or left them in the biosafety cabinet with the lights off. We measured each virus titer using 2–6 replicate samples by TCID_50_ or plaque assay. Data are represented as mean ± SD. Note. PFU, plaque forming units; R3, 3M panel respirator (1870+) FW, Type II EN 14683 generic face mask. The dotted line indicates the limit of detection.

**Fig. 4.**
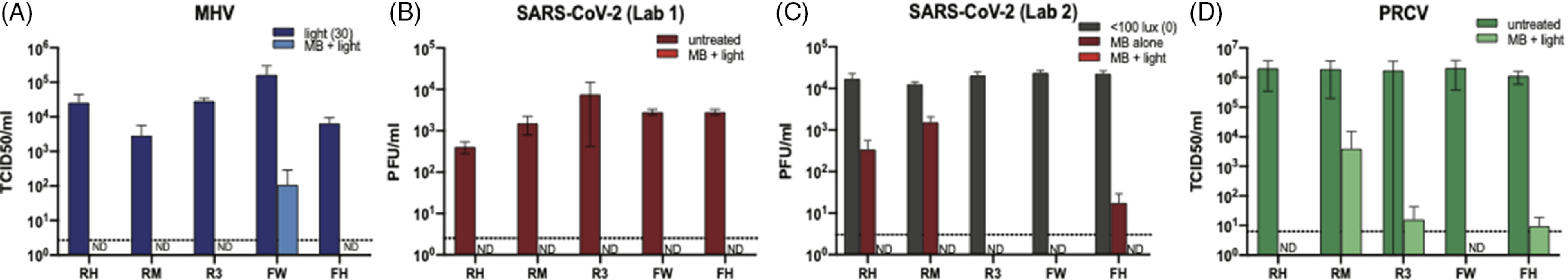
MBL inactivates MHV, SARS-CoV-2, and PRCV on multiple FFR and medical mask types. (A–C) We applied a 10-µL aliquot of SARS-CoV-2 or MHV to coupons of the indicated masks and dried them for 20 minutes. Depending on coupon size, we added 10–30 µL of 10 µM MB to each coupon and then treated the samples with light (50,000 lux) or protected them from light. (D) We injected 100 µL PRCV under the outer layer of intact FFRs or medical masks and allowed them to dry for 30 minutes. Subsequently, we sprayed the FFRs and medical masks with 10 µM MB and dried them for 30 minutes in the dark before exposure to red light (12,500 lux). We determined each virus titer using 2–6 replicate samples by TCID_50_ or plaque assay. Data are represented as mean ± SD. Note. PRCV, porcine respiratory coronavirus; MBL, methylene blue, and light; MHV, murine hepatitis virus; FFR, filtering facepiece respirator; MB, methylene blue; TCID_50_, median tissue culture infectious dose; ND, not detected; RH, Halyard duckbill respirator (Fluidshield-46727); RM, 3M half-sphere respirator (1860); R3, 3M panel respirator (1870+); FW, Type II EN 14683 generic face mask; FH, Type IIR ASTM F2100 Level 2 Halyard face mask. The dotted line indicates the limit of detection.

**Fig. 5.**
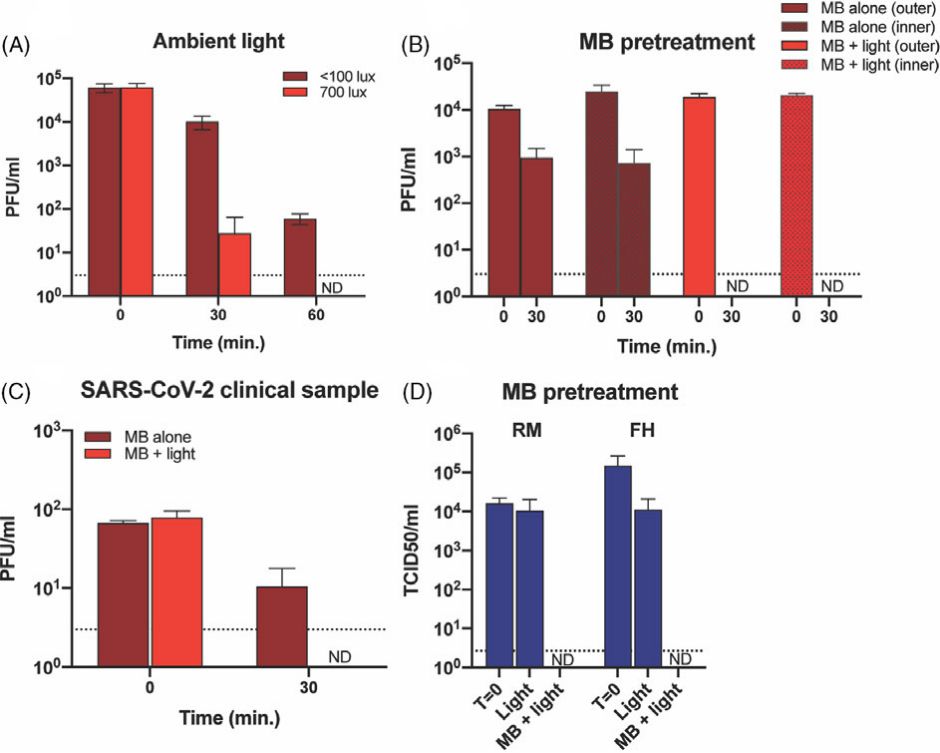
Potential applications of MBL in a clinical setting. (A) Effect of low light levels on SARS-CoV-2 inactivation using MB. We applied a 10-µL aliquot of SARS-CoV-2 to R3 coupons and dried them for 20 minutes. We added 10 µL of 10 µM MB to each coupon before treatment with 700 lux (the light level produced by the biosafety hood lights) or <100 lux of light. (B) Effect of MB pretreatment on SARS-CoV-2 inactivation. We cut coupons from R3 masks and soaked them for 1 hour in 10 µM MB. We then dried the coupons in the dark for 2 days before adding 10 µL virus to either the inner or outer layers. We exposed the samples to white light (50,000 lux) for 30 minutes and determined the virus titer by plaque assay. (C) Inactivation of a SARS-CoV-2 clinical specimen by MBL. We obtained a saliva specimen from a COVID-19 patient with a titer of 1.1 × 10^5^ PFU/mL for SARS-CoV-2. We applied 10 µL aliquots to coupons cut from an R3 mask, treated with 10 µM MB and exposed to white light (50,000 lux) for 30 minutes. We determined virus titer by plaque assay. (D) Effect of MB pretreatment on MHV inactivation using intact masks. We pretreated intact RM and FH masks with 10 µM MB by spraying the front and back with a total of 7–8 mL MB and allowed them to dry overnight in the dark. We inoculated the dried masks with MHV and exposed them to white light (50,000 lux) for 30 minutes. We then excised inoculated areas before elution and titration. We determined the virus titer by TCID_50_ assay. Note. ND, not detected; R3, 3M panel respirator (1870+); RM, 3M 1860 half-sphere respirator; FH, Type IIR Halyard face mask. The dotted line indicates the limit of detection.

**Fig. 6.**
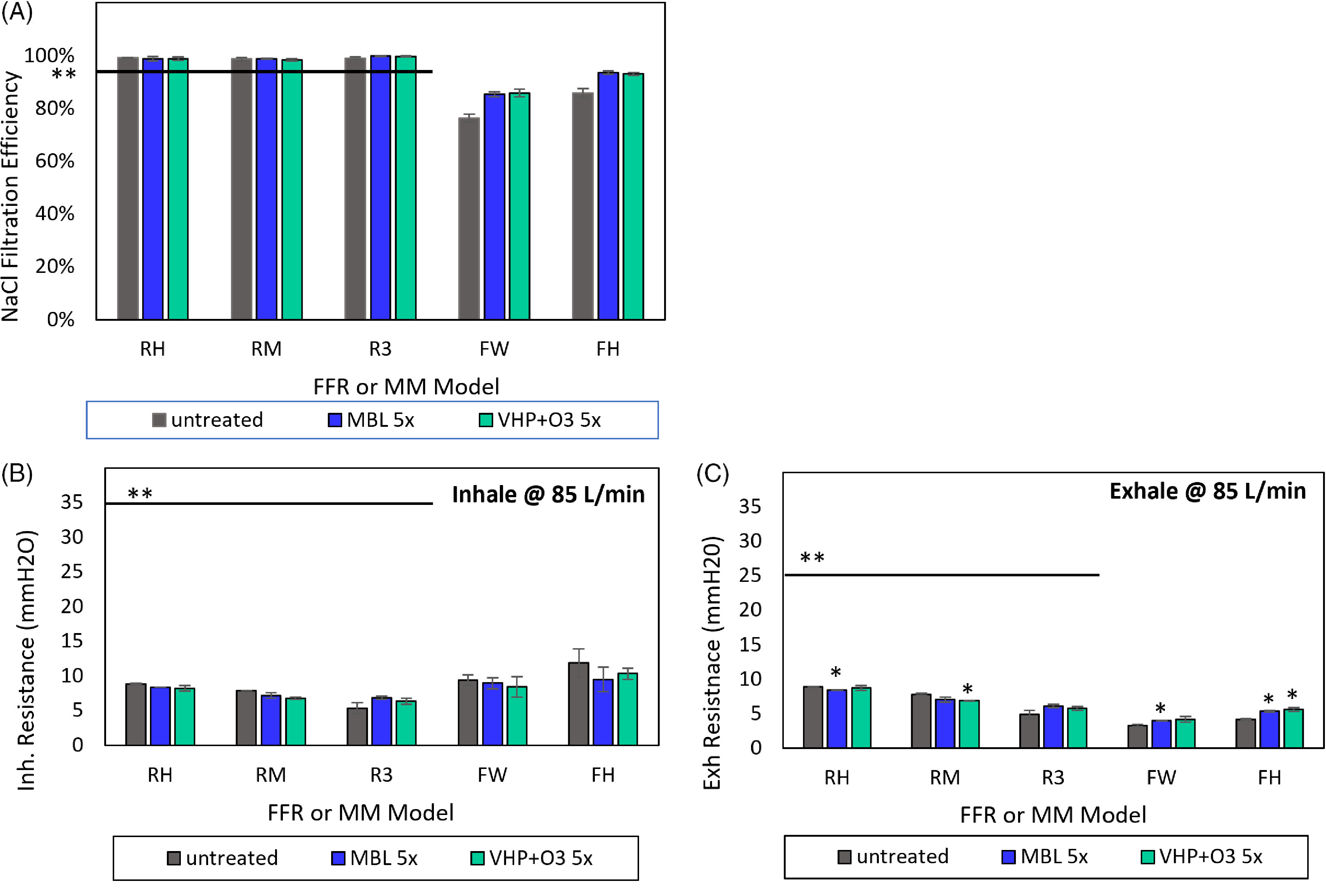
Effect of MBL and VHP+O_3_ treatments on NaCl submicron filtration efficiencies and breathability before and after 5 cycles of decontamination. (A) NaCl submicron filtration efficiency is a measure of the ability of an FFR or medical mask to capture aerosolized particles <1 µm, expressed as a percentage of particles that do not pass the material at a given velocity or flow rate. (B) Inhalation and (C) exhalation breathing resistances before and after 5 cycles of decontamination. The resistance to airflow during inhalation and exhalation is an indication of the difficulty in breathing through the respirators and masks. *Results from decontaminated FFRs and medical masks are significantly different from untreated masks (Student *t-test* or Mann-Whitney U test, *P* < .01). **Horizontal solid line in (A) represents the N95 filtration efficiency requirement of ≥95% particle filtration efficiency according to 42 CFR Part 84. Horizontal lines in (B) and (C) represent the following breathing resistance standards: inhalation: ≤35 mmH2O; exhalation: ≤25 mmH_2_O for respirators according to 42 CFR Part 84. EN 149 maximum inhalation resistance at 95 L/minute is 2.4 mbar, or ˜24 mmH_2_O. At a higher flow rate according to EN 149, the equivalent breathing resistance may increase slightly but can be similar to the 42 CFR Part 84 maximum inhalation resistance at 85 L/minute. Note. RH, Halyard duckbill respirator (Fluidshield-46727). RM, 3M half-sphere respirator (1860). R3, 3M panel respirator (1870+). FW, EN 14683 Type II generic face mask. FH, ASTM F2100 Level 2 Halyard face mask.

**Fig. 7.**
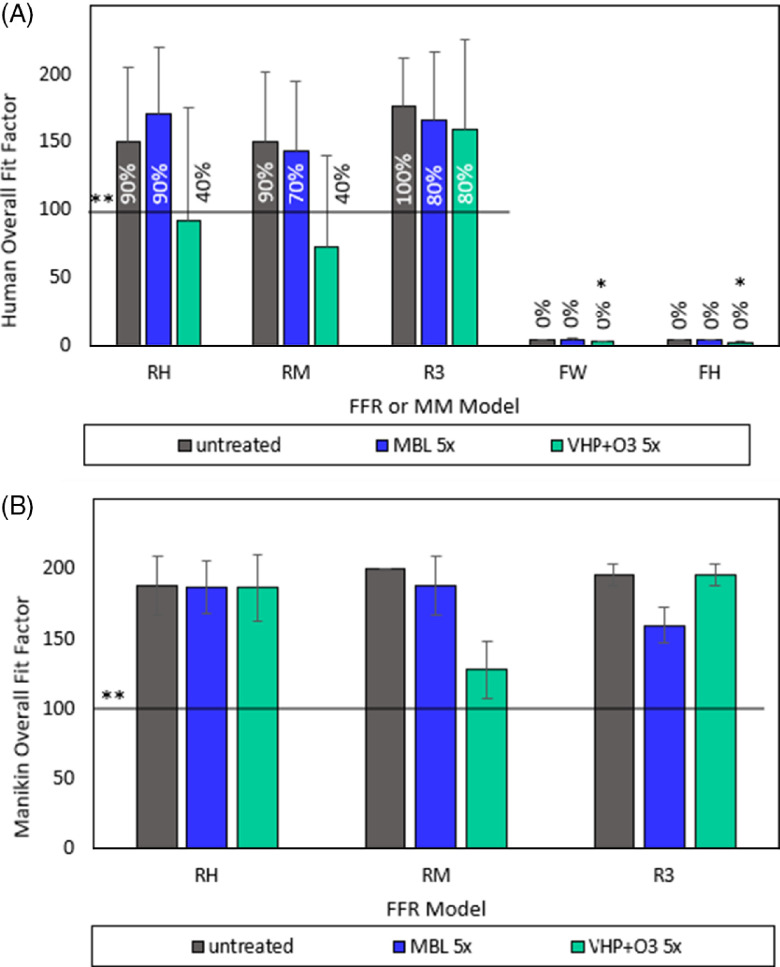
Effect of MBL and VHP+O_3_ treatments on human and manikin fit factor of FFRs and medical masks. (A) We performed human fit testing with volunteer participants who adjusted the FFRs and medical masks to achieve the highest fit factor or seal and subsequently performed head movements and remeasured fit or seal. (B) Manikin fit factors using advanced, realistic manikin headforms is a reproducible method to test fit without volunteer participants. We used the PortaCount PRO+ 8038 machine (TSI, Inc, Shoreview, MN) to determine the overall fit for both human participants and manikins headforms. *Indicates significantly different values between treated and untreated FFR or medical mask at *P* < .05, Student *t-test* or Mann-Whitney U test, as appropriate. **Horizontal line represents the following standard: Per OSHA 1910.134(f), if the overall fit factor as determined through an OSHA-accepted quantitative fit-testing protocol is ≥100 for tight-fitting half facepieces, then the fittest has been passed for that respirator. Percentages on or above each bar represent % of respirators or masks tested that surpassed this standard. Although the standard does not apply to face masks, we present the % to note the strong difference between respirator and face mask test results. Note. RH, Halyard duckbill respirator (Fluidshield-46727); RM, 3M half-sphere respirator (1860); R3, 3M panel respirator (1870+); FW, EN 14683 Type II generic face mask; FH, ASTM F2100 Level 2 Halyard face mask.

We observed that MBL specifically reduced SARS-CoV-2 infectivity with varying concentrations of MB when mixed with SARS-CoV-2 and exposed to 50,000 lux of white light. MB inhibited SARS-CoV-2 infectivity with a dose-dependent effect, with or without exposure to light. This virucidal effect was enhanced in the presence of light (Fig. 2B).

### MBL FFR and medical mask viral inactivation

When we cut coupons from a representative FFR (R3) or medical mask (Type II EN14 683 generic face mask, hereafter FW) and inoculated and treated them with MBL for the indicated periods, we observed that both viruses were sensitive to MBL treatment. Using 10 µM MB and light resulted in complete inactivation of SARS-CoV-2 and MHV on both FFR and medical mask materials after 5 minutes (Fig. 3). Using 1 µM MB, we observed complete inactivation after 30 minutes of light exposure, though we observed a 2–4 log viral titer reduction after 5 minutes. MB treatment in the absence of additional light also resulted in a substantial reduction of viral titers.

To ensure that MBL can efficiently decontaminate a variety of masks, we tested 3 more masks, including 2 additional FFRs, hereafter RH, and RM), and 1 additional medical mask (FH). We inoculated coupons or intact masks, treated with 10 µM MB and exposed to light for 30 minutes (Fig. 4A–D), conditions that demonstrated robust inactivation in the previous experiment. We observed complete inactivation (up to 4 log reduction) of SARS-CoV-2 for all respirators and masks tested. Treatment with MB without exposure to white light resulted in substantial virus reduction (Fig. 4C). We observed complete inactivation (4–5 log reduction) of MHV for FH, R3, RH, and RM masks. A low level of the virus was detectable in 1 replicate for FW (Fig. 4A). For PRCV, which was injected under the outer mask layer, we observed a >5-log virus reduction after treatment of FH, FW, R3, and RH masks. In contrast, we observed a 3-log reduction in RM (Fig. 4D). The overall percent reduction in virus titer after treatment across all FFRs/MMs and viruses ranged from 99.8% to >99.9% (Table 1). In addition, we tested the effect of MBL inactivation on FFR and medical mask straps inoculated with PRCV and noted a 2–4 log reduction in titers (Supplementary Fig. S4 online).

**Table 1.** Virus Reduction by 10 μM Methylene Blue with Light

	MHV		PRCV^b^		Lab 1 SARS-CoV-2		Lab 2 SARS-CoV-2		Avg. SARS-CoV-2		Overall Average	
Mask	Log Reduction	% Inactivation	Log Reduction	% Inactivation	Log Reduction	% Inactivation	Log Reduction	% Inactivation	Log Reduction	% Inactivation	Log Reduction	% Inactivation
RH	4.4	>99.9%	5.5	>99.9%	2.2	>99.4%	4.3	>99.9%	3.2	99.6%	4.1	99.8%
RM	3.5	>99.9%	2.7	99.8%	2.8	>99.8%	4.0	>99.9%	3.4	99.9%	3.2	99.9%
R3	5.1	>99.9%	5.0	>99.9%	3.3^a^	>99.9%	3.1	>99.9%	3.2	>99.9%	4.1	>99.9%
FW	5.8	>99.9%	5.5	>99.9%	3.6^a^	>99.9%	3.1	>99.9%	3.4	>99.9%	4.5	>99.9%
FH	3.8	>99.9%	5.1	>99.9%	3.1	>99.9%	4.2	>99.9%	3.6	>99.9%	4.0	>99.9%

Note. RH, Halyard duckbill respirator (Fluidshield-46727); RM, 3M half-sphere respirator (1860); R3, 3M Panel respirator (1870+); FW, Type II EN14 683 generic face mask; FH, Type II RASTM F2100 Level 2 Halyard face mask.

a Data extracted from inactivation curve.

b Light source for testing MB on PRCV = 12,500 lux red light. The light source for testing MB on all other virus types = 50,000 lux white light. Due to the limit of detection of the assays used to titer the virus, the highest % inactivation is indicated as >99.9%.

### Evaluation of potential applications of MBL in a clinical setting

We examined 3 potential applications of MBL in a clinical environment. First, because some clinical settings may not have access to bright light, we investigated whether 10 µM MB and ambient light would be sufficient to inactivate SARS-CoV-2. MB treatment and exposure to 700 lux (ambient light generated by light in a biosafety cabinet) for 60 minutes inactivated SARS-CoV-2 at nearly 5 log reduction. MB and <100 lux of light inactivated virus at almost 3 log reduction (Fig. 5A).

Second, we investigated the possibility of pretreating respirators or masks with MB.

After treating coupons with 10 µM MB, drying overnight, and inoculating them with SARS-CoV-2 on either the hydrophobic outer layer or the hydrophilic inner layer before exposure to 50,000 lux of white light for 30 minutes, we could not recover infectious virus from either side of the light-exposed coupons, signifying inactivation of >4 logs of virus (Fig. 5B).

We sprayed intact RM respirators and FH masks with 10 µM MB, dried them overnight, inoculated them with MHV, and exposed them to 50,000 lux of white light for 30 minutes. No viable virus was recovered (Fig. 5D).

Lastly, upon adding 10 µL of a clinical specimen (saliva) with a titer of 1.15 × 10^5^ PFU/mL obtained from a COVID-19 patient onto respirator coupons and treating them with 10 µM MB and white light (50,000 lux) for 30 minutes, we observed no viable virus after treatment. This result indicates the potential for this inactivation method in clinical settings in which viable virus may be protected by proteinaceous matrixes (Fig. 5C).

### Integrity testing

We employed standard test methods to determine whether MBL decontamination affected integrity, and we compared the results to those of the FDA-authorized VHP+O_3_ decontamination method. The following sections describe each of the integrity test methods and results (Supplementary Tables S2A–B online for complete results). Additional testing for medical masks included bacterial filtration efficiency, differential pressure, Sheffield dummy airflow differences, fluid (splash) resistance, and earloop integrity testing. Sheffield dummy airflow resistance and strap integrity testing were also conducted on FFRs (Supplemental Figs. S5–S8 online).

#### Filtration efficiency

Figure 6A depicts the filtration efficiency before (untreated) and after 5 cycles of decontamination with MBL and VHP+O_3_. We expected high filtration efficiencies for the FFRs because they are all NIOSH-approved N95 FFRs, which require ≥95% submicron filtration efficiency. All FFR models surpassed the minimum 95% filtration efficiency requirement before and after 5 cycles of decontamination using each method. Untreated FW and FH masks achieved 76% and 86% submicron filtration efficiency, respectively. Overall, MBL and VHP+O_3_ treatment of FFRs and medical masks did not cause any significant differences in the submicron filtration efficiency of the studied models (*P* > .01). Medical mask models continued to meet requirements of bacterial filtration efficiency after 5 cycles of decontamination (Supplementary Fig. S5 and Table S2A online).

#### Breathability

The resistance to airflow via inhalation and exhalation (breathability), is an indication of the difficulty in breathing through the respirators or masks. The FFR models achieved inhalation and exhalation resistances >60% below respective NIOSH 42 CFR Part 84 and EN 149 allowable maximum airflow resistance requirements after 5 cycles of decontamination (Fig. 6B, C). These resistance changes would not make it harder to breathe through the mask. Medical masks demonstrated similar inhalation resistances, and lower exhalation resistance values compared to FFRs after 5 cycles of decontamination. Both medical mask models were below their respective allowable maximum differential pressure limit after 5 cycles of decontamination (Supplementary Fig. S6 and Table S2A). Furthermore, we determined airflow differences of FFRs and medical masks using Sheffield Dummy simulated breathing (Supplementary Fig. S7 and Table S2A-B online). The MBL treatment did not affect the breathability of FFRs or medical masks, in terms of either inhalation and exhalation resistance or pressure drop.

#### Fluid resistance

We evaluated medical masks for their fluid (splash) resistance by challenging inside and outside of the masks with a small volume (∼2 mL) of a high-velocity stream of synthetic blood. In general, we observed that the decontamination process did not negatively impact the fluid resistance properties of the medical masks (Supplementary Fig. S8 and Table S2A online).

#### Fit testing

Fit testing measures how well a respirator or mask seals around the contours of the face. A good fit ensures that exchanged air is filtered through the respirator. Human fit testing demonstrated that respirators maintained quantitative fit values, or fit factors, above 100 after 5 cycles of decontamination. In contrast, VHP+O_3_ decontamination decreased RH fit and RM fit to the point of failure (Fig. 7A). Notably, 2 VHP+O_3_ decontamination cycles are the maximum authorized by FDA for N95 FFRs for the used system.^20^ We also performed human fit testing for the medical masks to demonstrate that these types of masks are not designed to ensure a tight fit (Fig. 7A). On some of the VHP+O_3_-treated FFRs and medical masks, human participants noted a “strong acrid odor” and some observed partial elasticity loss on treated straps and ear loops, and discoloration of the nosepiece foams (RM only). In contrast, some participants wearing the MBL-treated FFRs noted a “not unpleasant slight odor” at one of the fit testing sites. In addition to the discoloration, the nose bridge was more rigid for the 3 MBL-treated RMs.

We determined the manikin fit factor using an advanced, realistic manikin head form, which resulted in similar overall passing of OSHA criterion of 100 fit factor for all 3 FFRs (Fig. 7B).^16,21^ Observed differences between human and manikin fit can be attributed to testing procedure variability and structural facial variations. FFRs do not provide a universal fit for all wearers. Overall, MBL decontamination after 5 cycles of decontamination did not negatively impact integrity performance and fit while the VHP+O_3_ decontamination yielded some detrimental performance issues.

## Discussion

We have demonstrated that MB activated by white or red light effectively inactivates SARS-CoV-2 on FFR and medical mask surfaces and with a clinical specimen without affecting the integrity. MBL can be applied as a decontamination method for single-use FFRs and medical masks. Residual MB on the mask surface could potentially provide a novel means of continual inactivation of viral particles to decontaminate a mask while donned because MB inactivated SARS-CoV-2 on mask surfaces even under ambient light conditions.

For decades, MB has been recognized to have decontamination capabilities against a range of viral and bacterial pathogens,^7–9,22^ and MB is currently used to decontaminate plasma for transfusion^23^ and to sterilize convalescent serum for COVID-19 treatment.^10^

MBL is suitable for high- and low-resource settings because MB is inexpensive, globally available, and it does not require specialized equipment. Light sources can vary from white high-intensity lamps to ambient lighting to generate singlet oxygen (Supplementary Table S6 online). Future studies are warranted to investigate whether MBL could be used to inactivate additional pathogens and to decontaminate other forms of PPE such as gowns, gloves, and boots.

Our study has several limitations. We only tested a minority of FFR and medical mask models, yet there are ∼500 NIOSH-approved FFR models, and many others are used globally. MBL worked well on all FFRs and medical masks tested, except the “RM” FFR possibly owing to a distinct design characteristic that resulted in the variable outcome. In 2020, the CDC recommended that a decontamination method’s effectiveness be evaluated for specific FFR models in collaboration with the manufacturer, and if needed, a third-party laboratory.^24^ Although the N95 respirator reuse recommendation has been lifted in the United States, in other nations where PPE is reused, reusers may want to follow this guideline.

During our integrity testing, we did not simulate extended wear or multiple donnings and doffings, which could affect FFR fit and performance.^25,26^ In addition, off-gassing of MB or VHP+O_3_ was not evaluated. The biocompatibility of MB wearer inhalation was not tested, however, MB concentrations used were below those administered clinically (intravenously, orally, or intranasally).^7,27,28^ If the entire dose of 10 µM MB sprayed onto a mask was inhaled, which is unlikely, the total inhaled dosage would be 0.02 mg. The quantity of MB inhalation over time while wearing an MB-pretreated mask is under investigation.

We generalized our findings by demonstrating complete MBL inactivation employing the same methodology across multiple virology laboratories using 3 coronavirus species and a SARS-CoV-2 clinical sample. This signifies that emergent variants of SARS-CoV-2 would also be inactivated by MBL and that viruses requiring lower levels of biocontainment can be used for similar inactivation studies. Integrity tests in multiple testing centers using heterogeneous light administration methods reaffirms the reproducibility of our findings, and we replicated practical light scenarios expected in real-world settings.

In conclusion, MBL treatment inactivates SARS-CoV-2 on FFRs and medical masks without decreasing integrity and fit. Our findings provide a method for inexpensive, accessible, effective decontamination of PPE for reuse, applicable in high- and low-resource settings during supply shortages. Pretreatment of masks with MB could provide a novel means of continual decontamination reducing exposure to SARS-CoV-2.

## Supplementary material

For supplementary material accompanying this paper visit https://doi.org/10.1017/ice.2021.230. click here to view supplementary material
