# Supplementary material for: Addressing personal protective equipment (PPE) decontamination: Methylene blue and light inactivates severe acute respiratory coronavirus virus 2 (SARS-CoV-2) on N95 respirators and medical masks with maintenance of integrity and fit
Source: Infect Control Hosp Epidemiol. 2021 May 21;43(7):876–85. doi: 10.1017/ice.2021.230 (PMC8220024; doi:10.1017/ice.2021.230)
Supplement: Supplementary file 1 [file S0899823X21002300sup.zip › S0899823X21002300sup004.docx]

**Table S1: Respirator and Medical Mask Decontamination and Testing Matrix**

| **Viricidal Efficacy** |  |  | **Respirator or Medical Mask Type:** | **Halyard Duckbill Respirator (RH)** | **3M 1860 Half-sphere Respirator (RM)** | **3M 1870+ Panel Respirator (R3)** |  |  | **WHO Type II Face Mask (FW)** | **Halyard Type IIR Face Mask (FH)** |
| --- | --- | --- | --- | --- | --- | --- | --- | --- | --- | --- |
| **Decontamination Site** | **Decontamination Method** | **Respirator Virucidal Test** | **Respirator Test site** |  |  |  | **Mask Virucidal Test** | **Mask Test Site** |  |  |
| N/A | Untreated after MHV application | Virucidal efficacy on partial mask | University of Washington / Seattle Children’s Hospital | 3 | 3 | 3 | Virucidal efficacy on partial mask | University of Washington / Seattle Children’s Hospital | 3 | 3 |
| University of Washington / Seattle Children’s Hospital | MBL after MHV application | Virucidal efficacy on partial mask | University of Washington / Seattle Children’s Hospital | 3 | 3 | 3 | Virucidal efficacy on partial mask | University of Washington / Seattle Children’s Hospital | 3 | 3 |
| N/A | Untreated after SARS-CoV-2 application | Virucidal efficacy on partial mask | George Washington University | 3 | 3 | 3 | Virucidal efficacy on partial mask | George Washington University | 3 | 3 |
| George Washington University | MBL after SARS-CoV-2 application | Virucidal efficacy on whole mask | George Washington University | 3 | 3 | 3 | Virucidal efficacy on whole mask | George Washington University | 3 | 3 |
| N/A | Untreated after SARS-CoV-2 application | Virucidal efficacy on partial mask | University of Alberta | 3 | 3 | 3 | Virucidal efficacy on partial mask | University of Alberta | 3 | 3 |
| University of Alberta | MBL after SARS-CoV-2 application | Virucidal efficacy on partial mask | University of Alberta | 3 | 3 | 3 | Virucidal efficacy on partial mask | University of Alberta | 3 | 3 |
| N/A | Untreated after PRCV application | Virucidal efficacy on partial mask | University of Liège | 3 | 3 | 33 | Virucidal efficacy on partial mask | University of Liège | 31 | 3 |
| University of Liège | MBL after PRCV application | Virucidal efficacy on partial mask | University of Liège | 3 | 3 | 3 | Virucidal efficacy on partial mask | University of Liège | 3 | 3 |
|  |  |  |  |  |  |  |  |  |  |  |
|  | | | **Virucidal Efficacy Subtotal:** | **24** | **24** | **54** |  |  | **52** | **24** |
| **Integrity Testing** |  |  |  |  |  |  |  |  |  |  |
| **Decontamination Site** | **Decontamination Method** | **Respirator Integrity Test** | **Respirator Test Site** |  |  |  | **Medical Mask Integrity Test** | **Medical Mask Test Site** |  |  |
| N/A | untreated | N/A | N/A | N/A | N/A | N/A | Bacterial Filtration Efficiency and Pressure Drop; Fluid Penetration; Tensile Strength | Centexbel | 20 | 20 |
| University of Liège | MBL 5x | N/A | N/A | N/A | N/A | N/A | Bacterial Filtration Efficiency and Pressure Drop; Fluid Penetration; Tensile Strength | Centexbel | 20 | 20 |
| N/A | untreated | Human Fit (PortaCount) | University of Calgary | 5 | 5 | 5 | N/A | N/A | N/A | N/A |
| University of Calgary/ Alberta Health Services | MBL 5x | Human Fit (PortaCount) | University of Calgary | 5 | 5 | 5 | N/A | N/A | N/A | N/A |
| University of Calgary/ Alberta Health Services | VHP+O_3_ 5x | Human Fit (PortaCount) | University of Calgary | 5 | 5 | 5 | N/A | N/A | N/A | N/A |
| N/A | untreated | NaCl Filtration Efficiency and Breathing Resistance; Advanced Headform (Manikin); Tensile Strength (respirators and masks) | NIOSH NPPTL | 10 | 10 | 10 | NaCl Filtration and Breathing Resistance; Bacterial Filtration Efficiency and Pressure Drop; Fluid Penetration | Nelson Laboratories | 20 | 20 |
| Nelson Laboratories | MBL 5x | NaCl Filtration Efficiency and Breathing Resistance; Advanced Headform (Manikin); Tensile Strength (respirators and masks) | NIOSH NPPTL | 13 | 13 | 13 | NaCl Filtration and Breathing Resistance; Bacterial Filtration Efficiency and Pressure Drop; Fluid Penetration | Nelson Laboratories | 20 | 20 |
| Stryker Ltd. | VHP+O_3_ 5x | NaCl Filtration Efficiency and Breathing Resistance; Advanced Headform (Manikin); Tensile Strength (respirators and masks) | NIOSH NPPTL | 13 | 13 | 13 | NaCl Filtration and Breathing Resistance; Bacterial Filtration Efficiency and Pressure Drop; Fluid Penetration | Nelson Laboratories | 20 | 20 |
| N/A | untreated | Sheffield Dummy Fit | BSI LLC | 3 | 3 | 3 | Sheffield Dummy Fit | BSI LLC | 3 | 3 |
| Nelson Laboratories | MBL 5x | Sheffield Dummy Fit | BSI LLC | 3 | 3 | 3 | Sheffield Dummy Fit | BSI LLC | 3 | 3 |
| Stryker Ltd. | VHP+O_3_ 5x | Sheffield Dummy Fit | BSI LLC | 3 | 3 | 3 | Sheffield Dummy Fit | BSI LLC | 3 | 3 |
| N/A | untreated | Human Fit (PortaCount) | Stanford University | 5 | 5 | 5 | Human Fit (PortaCount) | Stanford University | 5 | 5 |
| 4C Air Inc. | MBL 5x | Human Fit (PortaCount) | Stanford University | 5 | 5 | 5 | Human Fit (PortaCount) | Stanford University | 5 | 5 |
| Stryker Ltd. | VHP+O_3_ 5x | Human Fit (PortaCount) | Stanford University | 5 | 5 | 5 | Human Fit (PortaCount) | Stanford University | 5 | 5 |
|  | | | **Integrity Subtotal:** | **75** | **75** | **75** |  |  | **124** | **124** |
|  |  |  |  |  |  |  |  |  |  |  |
|  |  |  | **GRAND TOTAL** | **99** | **99** | **129** |  |  | **176** | **148** |
