# Supplementary material for: Addressing personal protective equipment (PPE) decontamination: Methylene blue and light inactivates severe acute respiratory coronavirus virus 2 (SARS-CoV-2) on N95 respirators and medical masks with maintenance of integrity and fit
Source: Infect Control Hosp Epidemiol. 2021 May 21;43(7):876–85. doi: 10.1017/ice.2021.230 (PMC8220024; doi:10.1017/ice.2021.230)
Supplement: Supplementary file 1 [file S0899823X21002300sup.zip › S0899823X21002300sup003.zip!/ICE-2100230_Supplementary_Files/Lendvay_SUPPLEMENT_June_4_2021.docx]

**TABLE OF CONTENTS**

**1. Supplemental Material**

**1.A. Background: Methylene Blue as a Photosensitizer**

**1.B. Singlet Oxygen Production Rate - Theoretical Evaluation**

**2. Supplemental Results**

**2.A. Respirator and Mask Strap Viral Inactivation Testing**

**2.B. Bacterial Filtration Efficiency (BFE) using 3-micron Droplets Containing Bacteria**

**2.C. Breathability (Differential Pressure)**

**2.D. Simulated Wear and Air Flow Difference Detection using Sheffield Dummy**

**2.E. Fluid Resistance Testing**

**2.F. Tensile Testing of Elastomeric Straps and Ear Loops**

**3. Supplemental Materials and Methods**

**3.A. Respirators and Masks**

**3.B. Viruses**

**3.C. Cells for Tissue Culture**

**3.D. Virus Inoculation and Elution**

**3.E. Methylene Blue Treatment**

**3.F. Light Sources for Methylene Blue Testing**

**3.G. PPE Integrity Testing**

**3.H. Filtration Efficiency Testing**

**3.I. Breathability Testing**

**3.I.a. Sheffield AFD Testing**

**3.J. Fluid Resistance Testing**

**3.K. Fit Testing**

**3.K.a. Human Fit**

**3.K.b. Manakin Fit**

**3.K.c. Tensile Strength Testing**

**3.L. Statistical Analysis**

**4. Supplemental References**

**1. SUPPLEMENTAL MATERIAL**

**1.A. Background: Methylene Blue as a Photosensitizer**

Methylene blue (MB) is a positively charged phenothiazinium dye first synthesized in 1876 for use as a textile dye (1). The blue color of MB results from a strong absorption band in the red visible light spectrum with a peak at 664 nanometers as illustrated in [FIGURE S1] (ε_664_ = 85,000 M^-1^ cm^-1^)(2). Absorption of light by MB generates singlet oxygen (^1^O_2_) and other reactive species, which are microbicidal, a process known as photodynamic therapy (1,2). The initial step corresponds to the excitation of MB into its singlet excited state upon absorption of a photon, which is followed by an efficient intersystem crossing to the molecule’s triplet excited state (triplet quantum yield, Φ_T_ = 0.52) (2). The latter generates singlet oxygen almost quantitively via energy transfer (singlet oxygen quantum yield Φ _Δ_ ~ 0.50) (3). Alternatively, a small portion of the triplet excited state is deactivated via electron transfer to a reducing agent, which leads ultimately to the formation of a superoxide anion [FIGURE S2] (2).

***FIGURE S1:*** *Normalized absorption spectrum of a 10 μM solution of methylene blue (MB) (blue trace) in DI water recorded using a Cary 50 spectrophotometer (Agilent) using a quartz cuvette (1cm pathlength) with an overlay of the normalized irradiance spectrum of each light source used in this study. The Husky lamp irradiance spectrum (W/m^2^, black dashed trace) was recorded using a Stellar-RAD spectroradiometer (StellarNet Inc.); whereas the red lamp irradiance spectrum (W/m^2^, red dotted trace) was measured via a EKO Wiser spectroradiometer equipped with a MS-711 sensor. The chemical structure of MB is shown at the top of the figure.*

***FIGURE S2: Photosensitized production of ^1^O_2_ by Methylene Blue (MB).*** *Upon absorption of a photon (hν), methylene blue (MB) is promoted to one the of the vibrational levels of the molecule’s singlet excited state. The latter deactivates rapidly and returns to the lowest vibrational level of the first excited state (^1^MB). From there, the excited molecule will lose its energy by a combination of both radiative and non-radiative processes. IC corresponds to internal conversion, a non-radiative process, while F refers to fluorescence, a radiative process and ISC stands for intersystem crossing, another non-radiative process. Once ^1^MB intersystem cross to its triplet excited state (^3^MB), it can go back to the ground state non-radiatively via intersystem crossing or radiatively by emission of light referred to as phosphorescence (P). Alternatively, ^3^MB can transfer its energy to surrounding molecular oxygen (^3^O_2_) resulting in the formation of singlet oxygen (^1^O_2_).*

Singlet oxygen is the lowest excited state of molecular oxygen and can be viewed as an “energized” form of oxygen (4). Because it is an excited state species, singlet oxygen has a finite lifetime, which is environment-dependent. In water, singlet oxygen has a very short lifetime of ~3 μs (4) while in ambient air it can last up to 90 ms, thus creating different spheres of reactivity from hundreds of nanometers in aqueous environment to a few millimeters in air (2-5). However, if left unreacted, singlet oxygen simply reverts back to molecular oxygen (4). On the other hand, the reaction of singlet oxygen with biomolecules can lead to formation of secondary reactive species extending the microcidal effects of photodynamic therapy (6,7).

MB, without light activation, is approved by the U.S. Food and Drug Administration for treatment of acquired methemoglobinemia in children and adults by intravenous injection, at a dose of 1 mg/kg bodyweight followed by a second similar dose as needed. Topical application of MB in the mouth or nose followed by light activation is approved for use as a photodynamic microbicidal therapy for periodontitis in Canada, the United Kingdom, and the European Union, and for pre-operative intranasal disinfection in Canada. (8,9) MB and light is also routinely used commercially in Europe, South America, and Asia Pacific regions for viral disinfection of pooled human donor plasma. (10) MB is listed as one of the WHO’s Model List of Essential Medicines 2019. (11) MB is also widely used in a variety of off label indications including:

- Identification of lymph nodes for sentinel node biopsy after subcutaneous injection (12)
- As a marker dye for Barrett’s esophagus and colonic polyps during endoscopic evaluation (13,14)
- As an injection to identify ureteral leakage from trauma and the existence of pelvic fistulas (15,16)
- Treatment of ifosfamide neurotoxicity and cyanide poisoning (17)
- To evaluate aspiration of oral contents into the lung (18)
- In clinical trials as a vasopressor for septic shock, as cognitive enhancer for Alzheimer’s disease, as an oral treatment for malaria, and as a topical treatment for hidradenitis suppurativa (17)

One of the first studies demonstrating an antiviral effect of MB and light against a herpes virus was published in 1933 by Perdrau (19). Subsequent mechanisms of action research has shown that the photodynamic generation of singlet oxygen by light-activated MB attacks lipids, proteins, and nucleic acids, and has been demonstrated in multiple studies to inactivate a range of DNA and RNA viruses, including Ebola virus, Middle East Respiratory Syndrome Coronavirus, and more recently, SARS-CoV-2 (10, 20, 21) [TABLE S3].

***TABLE S3. Viral log reductions achieved by MB photodynamic therapy.***

| Reference | Pathogen | Log Reduction |
| --- | --- | --- |
| (10) |  |  |
|  | *Enveloped viruses*  Human immunodeficiency virus-1 (HIV-1) | ≥5.45 |
|  | West Nile Virus (WNV) | ≥5.78 |
|  | Bovine viral diarrhea virus (BVDV) | ≥5.44 |
|  | Pseudorabies virus (PRV) | ≥5.48 |
|  | Duck hepatitis B virus (HBV) | ≥6 |
|  | Influenza H3N2 | ≥4.40 |
|  | Cytomegalovirus (CMV) | ≥4.08 |
|  | Infectious bronchitis virus (IBV) | ≥4.90 |
|  | Hog cholera | ≥5.92 |
|  | Herpes simplex virus | ≥5.50 |
|  | Bovine herpes | ≥8.11 |
|  | Semliki Forest virus | ≥7.00 |
|  | Sindbis virus | ≥9.73 |
|  | Influenza virus | ≥5.1 |
|  | Vesicular stomatitis virus (VSV) | ≥4.89 |
|  | *Non-enveloped viruses*  Human adenovirus-5 (Had-5) | ≥5.33 |
|  | Calicivirus | ≥3.9 |
|  | Simian virus 40 (SV40) | ≥4 |
|  | Parvovirus B19 | ≥5 |
|  | Porcine parvovirus | ≥0 |
|  | Poliovirus | ≥1 |
|  | Hepatitis A | ≥0 |
| (20) |  |  |
|  | Ebola | ≥4.7 (light dose 30 J/cm^2^) |
|  | Middle East respiratory syndrome coronavirus (MERS-CoV) | ≥3.3 (light dose 30 J/cm^2^) |
| (21) |  |  |
|  | Severe acute respiratory syndrome coronavirus (SARS-CoV) | ≥3.1 (light dose 30 J/cm^2^) |
|  | Crimean–Congo hemorrhagic fever virus (CCHFV) | ≥3.2 (light dose 30 J/cm^2^) |
|  | Nipah virus (NiV) | ≥2.7 (light dose 30 J/cm^2^) |

**1.B. Singlet Oxygen Production Rate - Theoretical Evaluation.**

The fraction of photon (P) absorbed by one molecule per unit of time is given by the following integrated product:

$P= \int_{\lambda_{1}}^{\lambda_{2}} f\left( \lambda\right) \sigma\left( \lambda\right) d\lambda$	(eq 1)

where f(λ) is the photon flux (#photon/cm^2^/s) and σ(λ) corresponds to the absorption cross-section of the molecule (cm^2^).

The photon flux, f(λ), is obtained from the irradiance spectrum of the light source, as the irradiance I(λ) in W/cm^2^ (= J/s/cm^2^) corresponds to:

$I\left( \lambda\right)=f\left( \lambda\right)\times E(\lambda)$	(eq 2)

where E(λ) is the energy in Joules (J) of a photon of wavelength λ.

We recorded the irradiance spectrum of the Husky lamp using a Stellar-RAD spectroradiometer (StellarNet Inc.) and shown in [FIGURE S3]. In this figure, the spectroradiometer was located at a distance where the total illuminance was 100,000 lux.

***FIGURE S3:*** *Irradiance spectrum of the Husky light source measured at a distance that generates a total illuminance of 100,000 lux.*

Similarly, we recorded the irradiance spectrum of the Husky light source when the spectroradiometer was located at a distance where the total illuminance was 50,000 lux. Finally, we measured the irradiance spectrum of fluorescent light bulbs in a room under normal lighting condition at a total illuminance of 1300 lux.

We obtained the absorption cross section of one molecule of MB, σ(λ), from the knowledge of the molecule’s molar absorption coefficient, ε(λ), as per equation 3:

$\sigma\left( \lambda\right)= \frac{2.3\times{10}^{3}}{N_{A}} \varepsilon\left( \lambda\right)=3.824\times{10}^{-21} \varepsilon(\lambda)$	(eq. 3)

Therefore, under our experimental conditions, we calculated the fraction of photon (P) absorbed by one molecule of MB in 1 second to be 0.86 when the light source is a Husky lamp with a total illuminance of 100,000 lux. The fraction of photon (P) absorbed by one molecule of MB in 1 second becomes 0.56 when the total illuminance of the Husky lamp is reduced to 50,000 lux; whereas it is calculated to be 0.2 under ambient light (fluorescent light source) conditions with a total illuminance of 1300 lux. These data are summarized in [TABLE S4].

***TABLE S4*:** Calculated fraction of photon absorbed per molecule of MB per second (P) for various light sources.

|  | Husky LED  (100,000 lux) | Husky LED  (50,000 lux) | Fluorescent light  (1300 lux) |
| --- | --- | --- | --- |
| P | 0.86 | 0.56 | 0.2 |

The number of molecules of MB present on the total external surface of the mask can be obtained from knowledge of the initial concentration of MB and the total volume sprayed. If a total volume of 8 ml of MB solution is applied onto the surface of the mask in 6 sprays, 4 on the outside and 2 on the inside, then 5.33 ml are used to cover the outside surface of the mask. Therefore, 3.21×10^16^ molecules of MB will be present on the external surface of the mask for an initial concentration in MB of 10μM, while this number reduces to 3.21×10^15^ molecules for an initial concentration of MB of 1μM.

If we assume that all photons emitted by the light source are absorbed by the surface of the mask, then by taking into consideration the number of molecules of MB present on the external surface of the mask and the fraction of photon absorbed per molecule per second, we can easily obtain the total number of photons absorbed per second. The latter are summarized in [TABLE S5] for the different light sources.

***TABLE S5:*** Total number of photons absorbed per second by the external surface of the mask.

|  | Husky LED  (100,000 lux) | Husky LED  (50,000 lux) | Fluorescent light  (1300 lux) |
| --- | --- | --- | --- |
| [MB] = 10μM | 2.8×10^16^ | 1.8×10^16^ | 6.4×10^15^ |
| [MB] = 1μM | 2.8×10^15^ | 1.8×10^15^ | 6.4×10^14^ |

Since the singlet oxygen quantum yield (Φ_Δ_) represents the number of singlet oxygen molecule generated per number of photons absorbed by the photosensitizer, and MB has a Φ_Δ_ of 0.5, then it is estimated that at least 3.2×10^14^ of singlet oxygen molecules are produced per second for the entire external surface of the mask under ambient light irradiation. We summarized the number of singlet oxygen molecules produced per second under different irradiation conditions in [TABLE S6].

***TABLE S6:*** Number of singlet oxygen molecules produced per second on the entire external surface of the mask.

|  | Husky LED  (100,000 lux) | Husky LED  (50,000 lux) | Fluorescent light  (1300 lux) |
| --- | --- | --- | --- |
| [MB] = 10 μM | 1.4×10^16^ | 9×10^15^ | 3.2×10^15^ |
| [MB] = 1 μM | 1.4×10^15^ | 9×10^14^ | 3.2×10^14^ |

**2. SUPPLEMENTAL RESULTS**

**2.A. Respirator and Mask Strap Viral Inactivation Testing**

We inoculated FFR and MM straps/ear loops with 100µL of PRCV, dried for 30 minutes and then exposed to 10 µM MB with 30 minutes of light. We then cut out sections of the inoculated straps/ear loops and placed into 15mL Falcon tubes with MEM, and eluted any residual virus as with the mask material protocol. Inactivation noted in Supplemental FIGURE S4.

***FIGURE S4. PRCV Inactivation on inoculated FFR and MM straps and ear loops by MBL.*** *100 µl of PRCV was added onto both straps/ear loops of MMs, FFRs. The straps were allowed to dry for 30 minutes before exposure to 10 µM MB + light (30 min).* *Six inoculated, decontaminated straps (n=6) were analyzed in parallel to inoculated, untreated, positive control straps (n=6).* *Subsequently viral titers were determined by TCID_50_ assay. Data is represented as mean +/- SD. FH= Type IIR Halyard face mask. FW= Type II generic face mask. RH= Halyard duckbill respirator. FW= Type II generic face mask. R3= 3M panel respirator (1870+). Dotted line represents the lower limit of detection.*

**2.B. Bacterial Filtration Efficiency (BFE) using 3-micron Droplets Containing Bacteria**

We determined the filtration efficiency of MM material using bacterial filtration efficiency (BFE) testing (29, 30). BFE should be ≥98% according to EN 14683 for Type II and ASTM F2100 for Level 2 MMs (29, 31). Both FW and FH masks achieved greater than 98% BFE before and after 5CD of the three different decontamination methods. [FIGURE S5]. Overall, we did not observe significant differences in the BFE values of any of the tested mask models after 5CD with the MBL and VHP+O_3_ treatments.

***FIGURE S5. Effect of MBL and VHP+O_3_ treatments on the bacterial filtration efficiency of MMs******.*** *Bacterial filtration efficiencies of FFRs/MMs before and after 5CD. Bacterial filtration efficiency is the effectiveness of medical face mask material in preventing the passage of aerosolized bacteria suspended in 3 µm droplets, expressed as the percentage that does not pass the mask material at a 28.3 L/min flow rate (similar to human breathing at a light workload). *Horizontal solid lines represent the bacterial filtration efficiency (3 µm droplet size) requirement of ≥98% according to EN 14683 Type II and IIR and ASTM F2100 Level 2 and Level 3 MMs. FH=ASTM F2100 Level 2 Halyard face mask. FW=EN 14683 Type II generic face mask.*

**2.C. Breathability (Differential Pressure)**

The differential pressure (pressure drop) test measures the differential air pressure on either side of the MM materials using a digital manometer and is a required test method according to ASTM F2100-19 and EN 14683:2019 (29, 30). It is also one of the required test methods by FDA and the European Medical Device Directive for clearance of MMs. As shown in [FIGURE S6], all of the MMs performed below the maximum allowed differential pressure values before and after 5CD. We noted a slightly higher pressure drop after decontamination with VHP+O_3_ for both MMs. Overall, we did not report any concerns in terms of breathability after the decontamination treatments. We performed an additional, modified breathability test using a Sheffield dummy that simulates breathing and wear.

*

**

**

DIfferential Pressure (Pa/cm2)

| MM Model | untreated | MBL 5x | VHP+O3 5x |
| --- | --- | --- | --- |
| FW | 29.09 | 29.09 | 32.3 |
| FH | 40.98 | 41.16 | 44.48 |

***FIGURE S6. Effect of MBL and VHP+O_3_ treatments on the breathability of FFRs/MMs.*** *Differential pressure results for MMs before and after 5CD. Pressure drop is a measure of the differential air pressure on either side of the medical mask. *Results from decontaminated FFRs/MMs are significantly different from untreated masks (Student’s t test or Mann-Whitney U test p<0.01). **Horizontal solid lines represent the maximum allowed differential pressure in the following standards: <40 Pa/cm^2^ according to EN 14683:2019 Annex C for Type II; <58.83 Pa/cm^2^ according to ASTM F2100 for Level 2. FH=ASTM F2100 Level 2 Halyard face mask. FW=EN 14683 Type II generic face mask.*

**2.D.** **Simulated Wear and Air Flow Difference Detection using Sheffield Dummy**

Simulated wear air flow differences (SW-AFD) using a Sheffield dummy head connected to a breathing circuit were used to mimic rates of breathing ranging from shallow (30 L/min) to deep breathing (85 L/min and 160 L/min). The maximum air flows detected were similar to pressure drop differences, but tested at four air flow rates. Note that inhalation and exhalation AFDs at 85 L/min, Figure S5b and S5c, were similar in values. At higher air flows AFDs were also higher. As seen in the [FIGURE S7], MBL treatment did not alter AFD with inhales and exhales.

**Inhale @ 85 L/min**

**Inhale @ 28.3 L/min**

**B**

**A**

Air Flow Change (L/m)

| FFR or MM Model | untreated | MBL 5x | VHP+O3 5x |
| --- | --- | --- | --- |
| RH | -0.27 | -0.29 | -0.29 |
| RM | -0.26 | -0.26 | -0.25 |
| R3 | -0.22 | -0.23 | -0.22 |
| FW | -0.18 | -0.19 | -0.18 |
| FH | -0.16 | -0.16 | -0.15 |

Air Flow Change (L/m)

| FFR or MM Model | untreated | MBL 5x | VHP+O3 5x |
| --- | --- | --- | --- |
| RH | -0.85 | -0.88 | -0.87 |
| RM | -0.79 | -0.78 | -0.74 |
| R3 | -0.66 | -0.68 | -0.65 |
| FW | -0.54 | -0.61 | -0.56 |
| FH | -0.43 | -0.47 | -0.44 |

**Exhale @ 160 L/min**

**Exhale @ 85 L/min**

**D**

**C**

Air Flow Change (L/m)

| FFR or MM Model | untreated | MBL 5x | VHP+O3 5x |
| --- | --- | --- | --- |
| RH | 0.8 | 0.83 | 0.84 |
| RM | 0.74 | 0.75 | 0.72 |
| R3 | 0.63 | 0.66 | 0.62 |
| FW | 0.51 | 0.55 | 0.52 |
| FH | 0.37 | 0.42 | 0.4 |

Air Flow Change (L/m)

| FFR or MM Model | untreated | MBL 5x | VHP+O3 5x |
| --- | --- | --- | --- |
| RH | 1.62 | 1.66 | 1.69 |
| RM | 1.49 | 1.46 | 1.46 |
| R3 | 1.24 | 1.28 | 1.22 |
| FW | 0.97 | 1.05 | 1.02 |
| FH | 0.71 | 0.81 | 0.76 |

***FIGURE S7.*** ***Effect of MBL and VHP+O_3_ treatments on the air flow rate differences measured with the Sheffield Dummy Head for three FFRs (R3, RH, RM), two MMs (FH, FW) during inhalation and exhalation.*** ***(A)****: Inhale 28.3 L/m Test* ***(B)*** *Inhale 85 L/m Test.* ***(C)*** *Exhale 85 L/m Test.* ***(D)*** *Exhale 160 L/m Test. Air flow rate changes were used to mimic rates of breathing ranging from shallow to deep breathing. RH= Halyard duckbill respirator (Fluidshield-46727). RM=3M half-sphere respirator (1860). R3=3M panel respirator (1870+). FH=ASTM F2100 Level 2 Halyard face mask. FW=EN 14683 Type II generic face mask.*

**2.E.** **Fluid Resistance Testing**

During a medical procedure, a blood vessel is occasionally punctured resulting in a high velocity stream of blood impacting a medical face mask. Fluid (splash) resistance testing is used to evaluate the resistance of medical face masks to penetration by a small volume (~2 mL) of a high-velocity stream of synthetic blood (29, 30). Medical face mask pass/fail determinations are based on visual detection of synthetic blood penetration on the reverse side. We assessed fluid resistance properties of MMs by both challenging from the inside (inner surface) to investigate their potential source control performance, and from the outside (outer surface) to investigate the protection from splash and sprays that can be encountered during surgeries, patient care, and large droplets (respiratory secretions) that may be generated by other people’s coughs or sneezes.

FW masks demonstrated 60% pass before treatment, and 80% and 40% pass when challenged from inside (observed outside) after MBL and VHP+O_3_ treatments, respectively [FIGURE S8]. The passing results increased to 100% after MBL or VHP+O_3_ treatments. We observed that the decontamination processes increased the fluid resistance of outside materials of both MM models. There were no significant differences between treated masks and untreated masks (Fisher’s exact test).

***FIGURE S8.* *Effect of MBL and VHP+O_3_ treatments on the fluid (splash) resistance properties of inside and outside surfaces of MMs.*** *Fluid resistance of masks was assessed using a high-velocity (120 mmHg pressure) stream of artificial blood (2 mL). Ten masks were tested per condition, with all masks passing at 100%, meaning fluid penetration was visually undetectable on the opposite side. Masks were either challenged from the inside* ***(A)*** *or the outside* ***(B)****,* *with fluid resistance/penetration assessed on the opposite side. FH=ASTM F2100 Level 2 Halyard face mask. FW=EN 14683 Type II generic face mask.*

**2.F. Tensile Testing of Elastomeric Straps and Ear Loops**

We examined the changes in the elastic recovery of the FFR straps and MM ear loops to determine changes in headband integrity due to the decontamination process. The straps of the VHP+O_3_ treated RMs were frayed in several locations and significant deterioration was observed. This was also reflected in the human fit testing. Straps of RMs also showed increases in force in both the top and bottom straps after VHP+O_3_. We did not observe any visual degradation of the straps of any other model/decontamination method. Inconsistent changes were shown between the top and bottom straps with MBL-treated RM. The RH (MBL and VHP+O_3_) showed decreases in recorded force for both the top and bottom straps. The R3 (MBL and VHP+O_3_) showed increases in recorded force for both the top and bottom straps. Also, the decontamination methods significantly impacted the elastic recovery of the ear loops of the FHs while VHP+O_3_ treatment significantly reduced both MMs (p<0.01).

**3. SUPPLEMENTAL MATERIALS AND METHODS**

**3.A. Respirators and Masks**

In this study, we tested three FFR models - Halyard Fluidshield 46727 duckbill respirator (RH, NIOSH Approval Number: 84A-7521) (O & M Halyard,), 3M 1860 half-sphere respirator (RM, NIOSH Approval Number: 84A-3844), and 3M 1870+ panel respirator (R3, NIOSH Approval Number: TC-84A-5726) (3M), and two MM models - generic EN 14683 Type II medical mask (FW), Halyard ASTM F2100 Level 2 procedure mask (FH) (O & M Halyard) [FIGURE S9]. The FFRs used in this study are surgical FFRs which are NIOSH-approved particulate respirators that have also been cleared by the FDA as medical devices. See Supplemental Table S1 for details on which labs and testing sites respirators and masks were sent to for decontamination and testing.

***FIGURE S9.*** *FFRs and MMs used in this study*

**3.B. Viruses**

We obtained SARS-CoV-2 from Dr. Darryl Falzarano (VIDO) (GISAID accession ID: EPI_ISL_425177) used in Lab 2 or from a patient at the George Washington University Hospital used in Lab 1 and that was propagated in Vero CCL-81 cells. Viral titers were determined by plaque assay and typically reached 1.0-6.6 x10^6^ PFU/mL. A SARS-CoV-2 clinical saliva specimen with a titer of 1.1 x 10^5^ PFU/ml was provided by Dr. John Conly from Calgary, Alberta with University of Calgary Conjoint Health Research Ethics Board approval (ID# REB20-0444). The recombinant murine hepatitis virus (MHV) stock (rA59-E-FL-M) was described previously (23). Porcine respiratory coronavirus (PRCV), a spike gene deletion mutant of transmissible gastroenteritis virus (TGEV) and a member of the *Alphacoronavirus 1* species (24,25), was used as a SARS-CoV-2 surrogate. PRCV strain 91V44 (26) was passaged three times on ST cells. Both MHV and PRCV were used as SARS-CoV-2 surrogates.

**3.C. Cells for Tissue Culture**

We cultured Murine Delayed Brain Tumor (DBT) cells in MEM supplemented with 1% GlutaMAX, 1% HEPES, 1% NEAA, 10% Tryptose Phosphate Broth and 10% Fetal Bovine Serum (FBS). We purchased Vero CCL-81 cells from ATCC and cultured in DMEM supplemented with 10% FBS, 1% GlutaMAX, 1% Pen/Strep, and 0.1% Amphotericin B. Alternatively, we cultured Vero CCL-81 cells in MEM supplemented with 2 mM L-glutamine, 1 mM Sodium Pyruvate, 1x NEAA, 1% Pen/Strep, 0.1% Amphotericin B, and 10% FBS. We cultured Vero E6 cells (ATCC) in DMEM supplemented with 10% FBS and 1% GlutaMAX. We maintained Swine testicular (ST) cells in MEM supplemented with 5% FBS, 1% sodium pyruvate, and antibiotics (100U/ml penicillin, 0.1 mg/ml streptomycin, 0.05 mg/ml gentamycin). All cells were grown at 37°C in a humidified incubator with 5% CO2.

**3.D. Virus Inoculation and Elution**

We cut FFRs and MMs into 7-10 mm^2^ coupons and inoculated with the maximum available titer of SARS-CoV-2 or MHV or PRCV. The volume of the inoculum for SARS-CoV-2 and MHV was 10 μL. We added virus to the outer layer of each coupon (or inner layer where specified) with a pipette and dried for 20 mins before inactivation treatments were initiated. For elution, we soaked coupons in media in a 1.5 ml microtube (MHV, SARS-CoV-2 experiments), then vortexed or rocked on an orbital rocker for 10-20 minutes. Alternatively, we injected 100 μl PRCV under the FFRs/MMs outer layer using an ultra-fine insulin needle. After decontamination, we cut 34 mm^2^ coupons from the masks, placed in a 15-ml tube containing media and vortexed. We quantified remaining infectious virus by TCID_50_ or plaque assays.

**3.E. Methylene Blue Treatment**

We obtained MB from Sigma (M9140), ThermoFisher Scientific (J60823) or American Regent (0517-0374-05). We prepared stock solutions using ultrapure distilled water. At the appropriate time, we added 10-30 μl of MB at the indicated concentrations to the coupons and exposed to 50,000 lux of broad-spectrum light or 12,500 lux of red light for varying time points. We sprayed intact inoculated masks with 7-8 ml of 10 μM MB and allowed to dry for 30 minutes protected from light before exposure to 12,500 lux of red light for 30 minutes. For dark control, we left coupons and masks in biosafety hood with the light off or covered by aluminum foil (<100 lux).

For the pre-treatment tests, we soaked R3 coupons with 10 µM MB for >1 hour and dried 2 days protected from light. We spotted SARS-CoV-2 on outer or inner mask layers, dried for 20 minutes before exposure to 50,000 lux of light for 30 min. We eluted virus from the coupons and quantified by plaque assay. Intact RM and FW received 6 sprays on the front and 2 in the back (8 ml total) and dried overnight. We added MHV to three points on the outer surface, dried for 20 minutes and exposed to 50,000 lux of light for 30 minutes. We excised the inoculated areas from the mask, eluted, and quantified by TCID_50_ assay.

**3.F. Light Sources for Methylene Blue Testing**

Light boxes developed at Colorado State University and used at Seattle Children’s Research Institute, University of Calgary, George Washington University, and Nelson Laboratories consist of a transparent acrylic shelf placed horizontally to support the objects to be disinfected, surrounded by two Husky LED light panels (model# K40187) on top and bottom [FIGURE S10].

***FIGURE S10. Two constructed light boxes used in the study. Left:*** *Husky Light Box.* *This light box designed and constructed by the Hackathon Team at Colorado State University is configured with one Husky LED light. The clear acrylic shelf is positioned at a distance where the Husky light applies 50,000 lux of illuminance to the area delineated by the orange tape as measured by a light meter. This light box can also be fitted with a second Husky LED at the bottom to apply light to both sides of masks or respirators placed on the shelf.* ***Right:*** *University of Liege designed light box. This light box contains 3 x 6 Roleadro 60W Culture Indoor IP65 LED Horticultural T5 Grow Lamps with dimmable light control providing red light (610-720 nm) and blue light (400-520 nm) spectrum of light.*

The distance between the light panels and the objects could be adjusted by displacing the shelf on the aluminum rails that comprise the supporting frame of the light box. Each of the two 4000K color temperature LED light panels consumed about 56 W of electric power and provided a white light power of 13.7 W measured at the illumination surface of the LEDs. The spectrally integrated band between 560-710 nm of light coming out of the LED panel surface band had a divergence leading to a power density distribution ranging from about 170 W/m^2^ at the center to 90 W/m^2^ at the edge of a 10” (25.4 cm) diameter circular at 6” (15 cm) from the LED panel surface. This corresponded to an in-band illuminance of 64,300 and 34,000 lux (lux= lumen/m2), respectively. Similar Husky LEDs were used at the University of Alberta but had a 3500K color temperature. We used a digital light meter (Latnex LM-50KL) and shims to identify locations where the illumination within the bio containment hood equaled 50,000 lux. We determined light intensity using a light meter (Latnex, model LM-50KL or Cooke, Model CK-CL400). The University of Liege and Centexbel used a horticultural light box for their studies containing 3 x 6 red/blue 60 W horticultural LED lamps (Roleadro Culture Indoor IP65 LED Horticultural T5 Grow). We used a light meter (DeltaOHM, Model HD2102.2) to determine light intensity .

**3.G. PPE Integrity Testing**

We tested FFRs/MMs as-received (untreated), with VHP+O_3_, and with MBL. All decontaminated FFRs/MMs were treated off-site (Nelson, 4CAir, University of Liege) before reaching the integrity labs for testing.

**3.H. Filtration Efficiency Testing**

We assessed filtration using NaCl sub-micron particles for FFRs and MMs; and using 3-micron droplets that contain bacterial challenge for MMs. Although medical face masks are not typically tested using NaCl sub-micron particles, performing the tests with this challenging-size particle allowed comparison of filtration performance between masks and respirators. Sub-micron particulate filtration efficiency is a measure of the ability of a respirator or medical mask to capture aerosolized particles smaller than one micron, expressed as a percentage of a known number of particles that do not pass the material at a given face velocity for flat samples or flowrate for whole article testing. We tested untreated and decontaminated respirators for filtration efficiency using a modified version of the NIOSH Standard Test Procedure (STP) TEB-APR-STP-0059 (27), using an Automated Filter Tester (CERTITEST®, Model 8130, TSI, Inc.) and according to the National Personal Protective Technology Laboratory (NPPTL) Decontaminated Respirator Assessment Plan (22). In sub-micron testing, charged-neutralized particle size ranges from 0.022–0.259 µm with a count median diameter of 0.075 ± 0.020 µm and a geometric standard deviation (GSD) of less than 1.86 to give a mass median aerodynamic diameter of 0.3 μm, and air flow is 85 L/min (which simulates inhalation at heavy workload). Respirator testing was completed at the NIOSH NPPTL. We evaluated results against NIOSH performance criteria according to 42 CFR Part 84 for FFR approvals (minimum 95% filtration efficiency) (28). We tested untreated and decontaminated MMs following the same method at Nelson Laboratories.

The bacterial filtration efficiency (BFE) test is a conventional method to measure filtration efficiency of medical face masks (29, 31). BFE was measured using the bacteria *Staphylococcus aureus* (diameter: 1 μm) as the challenge organism. A suspension of *S. aureus* was aerosolized using a nebulizer to give a challenge level of 1700–3000 colony-forming units (CFU) per test as specified by the ASTM F2101 method (31). The bacterial aerosol is a water droplet containing the bacteria and not an individual bacterial particle. The particles were not charge neutralized for testing. The aerosol sample was drawn through a test sample clamped into the top of a 6-stage Andersen sampler with agar plates for collection of the bacteria particles at a flow rate of 28.3 L/min for 1 min. The design of the 6-stage Andersen sampler is based on the human respiratory tract, where all airborne particles greater than 0.65 μm are classified aerodynamically. The flow rate of 28.3 L/min is similar to human breathing flow rate (at light workload) to obtain deposition of particles in different stages of the Andersen sampler. Aerosol droplets generated in this test range from 0.65 to 7 µm with a mean particle size of approximately 3.0 µm. As previously mentioned, we employed BFE on MMs only and performed on untreated and decontaminated MMs at Nelson Laboratories and Centexbel. We evaluated results against performance criteria according to EN 148683 Type II and ASTM F2100 and Level 2 medical masks (≥98% 3 µm bacterial droplet filtration efficiency) (29, 30).

The filtration efficiency of fibrous filter materials is controlled by factors including, aerosol charge, particle size distribution, face velocity and filter material charge. When NIOSH sub-micron and BFE test methods are compared, NIOSH approval testing is considered as a more stringent or worst-case method, because of the use of charge neutralized aerosol size close to the most penetrating particle size at relatively higher flow rate (face velocity), to produce maximum penetration or conservative filtration efficiency.

**3.I. Breathability Testing**

We assessed breathability using inhalation and exhalation breathing resistance measurements according to the NIOSH standard testing procedures following 42CFR Part 84 for the all of the FFRs and MMs and pressure drop measurements for MMs only (ASTM F2100 and EN 14683) (29,30). We used an additional breathability assessment using clause 7.16 of the EN 149 standard for European respirators and termed “Sheffield Dummy Air Flow Differences” or Sheffield AFD (EN 149).

We tested inhalation and exhalation resistance of devices using NIOSH STPs (TEB-APR-STP-0007 and TEB-APR-STP-0003). We recorded the results in mmH_2_O and evaluated against NIOSH performance maximum limits for FFR approvals (25 mmH_2_O for exhalation and 35 mmH_2_O for inhalation) at approximately 85 ± 2 L/min airflow. Respirator testing was completed at the NIOSH NPPTL while MMs were tested at the Nelson Laboratories.

The differential pressure (*delta p*) testing of untreated and decontaminated samples was performed according to ASTM F2100 and EN 14683 (29, 30) and measured the differential air pressure on either side of the mask using a digital manometer at Nelson Laboratories and Centexbel Laboratories. The *delta p* values were reported in mm water/cm^2^ (required units for ASTM F2100) and Pa/cm^2^ (required units for EN 14683) of test area and evaluated against ASTM F2100 Level 2 and EN 14683 Type II requirements for and masks (ASTM F2100 <6.0 mm H_2_O/cm^2^ and <40Pa/cm^2^ for Type II).

**3.I.a. Sheffield AFD Testing**

We used the simulated wear treatment set-up of clause 7.16 of the EN 149 standard to determine differences in air flow between untreated and decontaminated respirators and masks. Three simulated wear conditions mimicked shallow to deep breathing and at variations of conventional flow rates: inhalation at 28.3 L/min (EN 149 and EN 14683), inhalation at 85 L/min (NIOSH N95) and exhalation at 85 L/min (NIOSH N95) and exhalation at 160 L/min (EN 149). Since this modified method is not a requirement for mask or respirator certification, we used Sheffield AFD to detect differences caused by the decontamination process compared to untreated masks or respirators.

**3.J. Fluid Resistance Testing**

Testing the fluid penetration resistance (resistance to splash and spray) by synthetic blood is one of the requirements of FDA surgical mask clearance in the U.S. for all three different levels of masks defined in ASTM F2100. It is also required by EN 14683 for Type IIR masks only. We performed the synthetic blood penetration testing of untreated and decontaminated samples on MMs at Nelson Laboratories and Centexbel according to ASTM F1862 or ISO 22609:2004 at 120 mmHg pressure which is required for ASTM F2100 Level 2 masks and EN 14683 Type IIR masks (29, 30). For each mask type, we tested five samples with the outside facing the synthetic blood to determine the barrier resistance and five samples with the inside facing the synthetic blood to assess the source control properties. We calculated the percentage of passing samples (no visible penetration) and compared it to ASTM F2100 requirements (29 out of the 32 passing results).

**3.K. Fit Testing**

*3.K.a. Human Fit*

We conducted fit testing with the PortaCount® Pro+ 8038 at two study sites: Stanford University, and the University of Calgary. The human fit testing was exempt from ethics board review by both the Research Compliance Office, Stanford University (March 23, 2020) and the Conjoint Health Research Ethic Board, University of Calgary (June 12, 2020). We conducted all fit testing of FFRs with OSHA-approved protocols for testing N95. However, sample sizes and study designs differed between sites. Stanford University performed fit testing of test masks on a human model (LC) using the OSHA test protocol of the PortaCount Pro+ 8038. At the University of Calgary, three groups of five healthcare workers completed fit tests in a repeated-measures design, with a respirator type between-subjects factor. At both sites, the fit test protocol consisted of the dynamic tasks: regular breathing, heavy breathing, turning head side-to-side, moving head up-and-down, talking, bending over with regular breathing. Each cycle of tests was performed twice for each mask and Fit Factor (FF) was calculated.

In addition to these measures, participants at the University of Calgary (including 15 additional healthcare workers who participated in a separate study arm focused on surgical/procedure and cloth masks; total N = 30) provided data on measures related to the physical appearance, physical comfort, and trustworthiness and acceptance of the decontaminated masks. Data from these measures, which is not included in the current manuscript, will be reported separately in a future publication.

*3.K.b. Manikin Fit*

We conducted the static advanced headform fit testing (22) used to determine the anticipated changes in fit. Static fit testing was completed on FFRs only at NIOSH NPPTL using a static advanced headform (StAH) that quantifies the changes in manikin fit factor. The TSI, Inc. PortaCount® PRO+ 8038 (Shorewood, Minnesota, USA) in “N95 Enabled” mode was used for this evaluation. Assessments of respirators were done using normal and deep breathing without dynamic movements and without a speaking passage (22).

*3.K.c. Tensile Strength Testing*

We determined the changes in the elastic recovery of the respirator straps and mask ear loops to determine changes in strap integrity due to the decontamination process. We used the method defined in the National Personal Protection Technology Laboratory (NPPTL) Decontaminated Respirator Assessment Plan (22) for FFR straps and a slightly modified version for MM ear loops.

**3.L. Statistical Analysis**

We generated measurements with standard error bars using Prisma Analytics (Munich, Germany). We calculated means and standard deviations or percent pass of each integrity test method separately by FFR/MM style. We pooled the data for each integrity test method conducted at more than one test site to create overall means and standard deviations or percent pass. We tested normality of the data distribution using the Shapiro-Wilk test. We calculated significant differences between untreated and treated FFR/MM with Student’s t-tests, Mann-Whitney U tests, or Fisher’s exact tests, as appropriate (SAS v9.4). We calculated EC_50_s using GraphPad Prism 8. We presented full results of integrity tests in Supplemental Table S2A and 2B.

**4. Supplemental References**

1.	Wainwright M, Crossley KB. Methylene Blue - a Therapeutic dye for all seasons? *Journal of Chemotherapy* [Internet] 2002; 14:431–43. Available from: https://doi.org/10.1179/joc.2002.14.5.431

2.	Tardivo JP, Del Giglio A, de Oliveira CS, Gabrielli DS, Junqueira HC, Tada DB, et al. Methylene blue in photodynamic therapy: From basic mechanisms to clinical applications. *Photodiagnosis and Photodynamic Therapy* [Internet] 2005; 2:175–91. Available from: http://www.sciencedirect.com/science/article/pii/S1572100005000979

3.	Fernandez JM, Bilgin MD, Grossweiner LI. Singlet oxygen generation by photodynamic agents. *Journal of Photochemistry and Photobiology B: Biology* [Internet] 1997; 37:131–40. Available from: http://www.sciencedirect.com/science/article/pii/S1011134496073496

4.	Boix-Garriga E, Rodríguez-Amigo B, Planas O, Nonell S. Chapter 2 Properties of Singlet Oxygen. In: Singlet Oxygen: Applications in Biosciences and Nanosciences, Volume 1 [Internet]. *The Royal Society of Chemistry* 2016: 23–46. Available from: http://dx.doi.org/10.1039/9781782622208-00023

5.	Midden WR, Wang SY. Singlet oxygen generation for solution kinetics: clean and simple. *Journal of the American Chemical Society* [Internet] 1983; 105:4129–35. Available from: https://doi.org/10.1021/ja00351a001

6.	Ouédraogo GD, Redmond RW. Secondary reactive oxygen species extend the range of photosensitization effects in cells: DNA damage produced via initial membrane photosensitization. *Photochemistry and Photobiology* [Internet] 2003; 77:192–203. Available from: https://doi.org/10.1562/0031-8655(2003)0770192SROSET2.0.CO2

7.	Redmond RW, Kochevar IE. Spatially resolved cellular responses to singlet oxygen. *Photochemistry and Photobiology* [Internet] 2006; 82:1178. Available from: https://doi.org/10.1562/2006-04-14-IR-874

8.	Branson RD, Blakeman TC, Robinson BRH, Johannigman JA. Use of a single ventilator to support 4 patients: Laboratory evaluation of a limited concept. *Respiratory Care* 2012; 57:399–403.

9.	Joseph B, Janam P, Narayanan S, Anil S. Is antimicrobial photodynamic therapy effective as an adjunct to scaling and root planing in patients with chronic periodontitis? A systematic review. *Biomolecules* 2017; 7:79.

10.	Seghatchian J. Bio of theme Editor: Dr. J Seghatchian. Transfusion and Apheresis *Science* 2018; 57:308–9.

11.	Organización Mundial de la Salud (OMS). World health organization model list of essential medicines. *Mental and Holistic Health: Some International Perspectives* 2019; 21:119–34.

12.	Li J, Chen X, Qi M, Li Y. Sentinel lymph node biopsy mapped with methylene blue dye alone in patients with breast cancer: A systematic review and metaanalysis. *PLoS ONE* 2018; 13:1–18.

13.	Canto MI. Vital staining and Barrett’s esophagus. *Gastrointestinal Endoscopy* 1999; 49:S12-6.

14.	Buchner AM. The Role of Chromoendoscopy in evaluating colorectal dysplasia. *Gastroenterology & Hepatology* 2017; 13:336–47.

15.	More R. What is the role of indigo carmine or methylene blue in the intraoperative workup of ureteral injury? MedScape. 2018; 9–12.

16.	Hanash KA, Al Zahrani H, Mokhtar AA, Aslam M. Retrograde vaginal methylene blue injection for localization of complex urinary fistulas. *Journal of Endourology* 2003; 17:941–3.

17.	Koch R. Some Drugs and Herbal Products. IARC monographs on the evaluation of carcinogenic risks to humans. 2016; 108:7–419.

18.	Garuti G, Reverberi C, Briganti A, Massobrio M, Lombardi F, Lusuardi M. Swallowing disorders in tracheostomised patients: A multidisciplinary/multiprofessional approach in decannulation protocols. *Multidisciplinary Respiratory Medicine* 2014; 9:1–10.

19.	Perdrau JR, Charles T. The photodynamic action of methylene blue on bacteriophage. *Proc R Soc Lond* B.1933; 112277–287; http://doi.org/10.1098/rspb.1933.0010

20.	Eickmann M, Gravemann U, Handke W, Tolksdorf F, Reichenberg S, Müller TH, et al. Inactivation of Ebola virus and Middle East respiratory syndrome coronavirus in platelet concentrates and plasma by ultraviolet C light and methylene blue plus visible light, respectively. *Transfusion* 2018; 58:2202–7.

21.	Eickmann M, Gravemann U, Handke W, Tolksdorf F, Reichenberg S, Müller TH, et al. Inactivation of three emerging viruses – severe acute respiratory syndrome coronavirus, Crimean–Congo haemorrhagic fever virus and Nipah virus – in platelet concentrates by ultraviolet C light and in plasma by methylene blue plus visible light. *Vox Sanguinis* 2020; 115:146–51.

22.	NIOSH. Decontaminated Respirator Assessment Plan. NIOSH (2020a). 2020. https://www.cdc.gov/niosh/npptl/respirators/testing/pdfs/NIOSHApproved_Decon_TestPlan10.pdf (accessed 12/20/2020)

23	Boscarino JA, Logan HL, Lacny JJ, Gallagher TM. Envelope protein palmitoylations are crucial for murine coronavirus assembly. *Journal of Virology* [Internet] 2008; 82:2989–99. Available from: http://www.ncbi.nlm.nih.gov/pubmed/18184706

24.	Pensaert M, Callebaut P, Vergote J. Isolation of a porcine respiratory, non-enteric coronavirus related to transmissible gastroenteritis. *The Veterinary Quarterly* 1986; 8:257–61.

25.	Wesley RD, Hill HT, Biwer JD. Evidence for a porcine respiratory coronavirus, antigenically similar to transmissible gastroenteritis virus, in the United States. Vol. 2, *Journal of Veterinary Diagnostic Investigation* 1990; 2:312–7.

26.	Cox E, Hooyberghs J, Pensaert MB. Sites of replication of a porcine respiratory coronavirus related to transmissible gastroenteritis virus. *Research in Veterinary Science* 1990; 48:165–9.

27.	NIOSH. Determination of Particulate Filter Efficiency Level for N95 Series Filters against Solid Particulates for Non-Powered, Air-Purifying Respirators Standard Testing Procedure (STP). [Internet]. NIOSH 2019. Available from: https://www.cdc.gov/niosh/npptl/stps/pdfs/TEB-APR-STP-0059-508.pdf (accessed 12/20/2020)

28.	NIOSH. 2 CFR Part 84. NIOSH [Internet]. 2020. Available from: https://ecfr.io/Title-42/pt42.1.84 (accessed 12/20/2020)

29.	ASTM. F2100-19e1 Standard Specification for Performance of Materials Used in Medical Face Masks. ASTM International. West Conshohocken, PA; 2020.

30.	EN 14683:2019, Medical face masks. Requirements and test methods. https://www.nelsonlabs.com/wp-content/uploads/2018/07/Face-Masks-2019.pdf (accessed 12/20/2020)

31.	ASTM. F2101-19 Standard Test Method for Evaluating the Bacterial Filtration Efficiency (BFE) of Medical Face Mask Materials, Using a Biological Aerosol of Staphylococcus aureus. ASTM International. West Conshohocken, PA: ASTM International 2019.
